# Photodynamic Inactivation of Bacteria with Porphyrin Derivatives: Effect of Charge, Lipophilicity, ROS Generation, and Cellular Uptake on Their Biological Activity In Vitro

**DOI:** 10.3390/ijms21228716

**Published:** 2020-11-18

**Authors:** Adam Sułek, Barbara Pucelik, Marcin Kobielusz, Agata Barzowska, Janusz M. Dąbrowski

**Affiliations:** 1 Faculty of Chemistry, Jagiellonian University, 30-387 Krakow, Poland; adam.sulek@doctoral.uj.edu.pl (A.S.); marcin.kobielusz@uj.edu.pl (M.K.); 2 Małopolska Center of Biotechnology, Jagiellonian University, 30-387 Krakow, Poland; barbara.pucelik@uj.edu.pl (B.P.); agata.barzowska@doctoral.uj.edu.pl (A.B.)

**Keywords:** antimicrobial activity, efflux pumps, multidrug resistance (MDR), photodynamic inactivation (PDI), porphyrins, reactive oxygen species (ROS), singlet oxygen

## Abstract

Resistance of microorganisms to antibiotics has led to research on various therapeutic strategies with different mechanisms of action, including photodynamic inactivation (PDI). In this work, we evaluated a cationic, neutral, and anionic *meso*-tetraphenylporphyrin derivative’s ability to inactivate the Gram-negative and Gram-positive bacteria in a planktonic suspension under blue light irradiation. The spectroscopic, physicochemical, redox properties, as well as reactive oxygen species (ROS) generation capacity by a set of photosensitizers varying in lipophilicity were investigated. The theoretical calculations were performed to explain the distribution of the molecular charges in the evaluated compounds. Moreover, logP partition coefficients, cellular uptake, and phototoxicity of the photosensitizers towards bacteria were determined. The role of a specific microbial efflux pump inhibitor, verapamil hydrochloride, in PDI was also studied. The results showed that *E. coli* exhibited higher resistance to PDI than *S. aureus* (3–5 logs) with low light doses (1–10 J/cm^2^). In turn, the prolongation of irradiation (up to 100 J/cm^2^) remarkably improved the inactivation of pathogens (up to 7 logs) and revealed the importance of photosensitizer photostability. The PDI potentiation occurs after the addition of KI (more than 3 logs extra killing). Verapamil increased the uptake of photosensitizers (especially in *E. coli*) due to efflux pump inhibition. This effect suggests that PDI is mediated by ROS, the electrostatic charge interaction, and the efflux of photosensitizers (PSs) regulated by multidrug-resistance (MDR) systems. Thus, MDR inhibition combined with PDI gives opportunities to treat more resistant bacteria.

## 1. Introduction

Porphyrins, due to a strong visible absorption attributed to π–π* electronic transitions, play a crucial role in many metabolic and photochemical processes, including electron transfer [1], oxygen transport [2], and oxygenation catalysis [3,4]. Recent investigations on synthetic porphyrins have mainly focused on their photochemical activity, allowing their application in photodynamic therapy (PDT) and photodynamic inactivation of microorganisms (PDI) [5,6,7]. The photodynamic action of modified porphyrins is strongly dependent on the optical/photophysical properties, such as intense absorption in UV-Vis-NIR, the high quantum yield of intersystem crossing (ISC), and efficient energy transfer from the triplet excited state (T_1_) of these dyes to molecular oxygen with the formation of singlet oxygen (type II photochemical reaction) [8,9,10]. Type-I reactive oxygen species (ROS) generation processes based on photoinduced electron transfer are competitive to the type-II mechanism [11] and may significantly influence photosensitizer (PS) photoactivity [12,13,14,15]. The previous studies have demonstrated that halogenated tetrapyrrole derivatives indicate a significantly higher spin-orbital coupling constant than their unmodified analogues, making ISC more favorable [16,17,18,19,20]. The introduction of sulfonic groups allows to increase their solubility in water, without changing their spectroscopic parameters, and the additional substitutes act as steric hindrance, making the compound more stable [21,22]. Despite the increased number of available antiseptics and disinfectants, bacterial infection remains a major health problem, including dental plaque, dermal and oral infections, urinary tract infections, and implant- or catheter-associated infections [23,24,25,26,27,28]. The presence of antibiotic-resistant bacteria still requires research on other strategies to eradicate bacteria resistant to many drugs [29,30,31,32].

As an alternative approach to overcome bacterial resistance, PDI is gaining prominence in the last decade, especially for presenting a nonspecific mechanism of action with the receptor but relies on damage to the outer membrane and internal structures, such as the lipids, proteins, or nucleic acids, which minimizes the possibility of the emergence of resistance [4,33]. Porphyrin-based PSs used in anticancer PDT may also be applied in PDI due to their photochemical properties, particularly the ability to generate singlet oxygen. However, the structural factors affecting the subcellular localization of the photosensitizer are associated with (1) the total charge, which ranges from −4 to +4 for tetraphenyl derivatives; (2) the logP partition coefficient, which expresses the physicochemical nature of the compound; and (3) the location of the substitutes in a tetrapyrrole ring [34,35]. The current research on ideal PSs for PDI focuses on charged side groups, which connect to a bacteria wall through electrostatic interactions [29,36]. For instance, positively charged porphyrins strongly interact and accumulate in Gram-negative bacteria strains [37]. Moreover, (N-methylpyridinium-4-yl)-substituted PSs are also known from their direct binding to DNA [38]. The porphyrin ring modifications are undertaken to modulate both the redox properties and change the physicochemical features, charge distribution, and lipophilicity [39,40,41].

The relatively weak defense mechanisms in bacteria against singlet oxygen-induced damages also contribute to the high efficiency of PDI [42]. Therefore, it is still unclear which type of ROS is more effective: extracellular hydroxyl radicals damaging the bacterial wall, singlet oxygen disrupting intracellular structures, or a combination of both [43]. Efflux pumps have become broadly recognized as major microbial resistance components. Some of them selectively remove specific drugs, while others can mediate the efflux of structurally different molecules [44,45]. The resistance is often related to efflux pumps’ overexpression, such as P-glycoprotein (P-gp)—a family of ATP-binding cassette (ABC) transporter proteins responsible for pumping out exogenous molecules from cells. As P-gp actively removes drugs out of bacteria, the intracellular concentration of the drugs/PSs dramatically reduces. Porphyrin uptake and efflux seem to be regulated by the TolC system in *E. coli* [46]. In *S. agalactiae*, two co-regulated efflux transporters modulate intracellular heme and protoporphyrin IX availability. In contrast, the PDT pattern of amphiphilic protoporphyrin diarginate (PPArg) in a variety of efflux *S. aureus* strains show no significant correlation for the PS with MES [47]. One representative study has shown that efflux pump receptors reduce efflux pump activity using an MDR modulator—verapamil. The concept of antimicrobials’ synergistic action with efflux pump inhibitors (EPIs) has also been exploited in PDT to potentiate the photodynamic action of PSs. *S. aureus* containing NorA exhibit resistance to many compounds, including quaternary ammonium compounds, such as verapamil, and the dyes ethidium bromide, rhodamine, and acridines [48,49]. However, small-molecule efflux pump inhibitors substantially enhanced the photodynamic effect mediated by methylene blue or toluidine blue O in *S. aureus*, which affect NorA, by reducing the viable cells and fluorescent dye accumulation [50,51].

In this paper, we have put forward the hypothesis of whether polarity-tunable porphyrin derivatives could potentially act as substrates of microbial MDR. The research is focused on two main problems: (1) the elucidation of photogenerated ROS mechanisms using electrochemical measurements supported by theoretical calculations and detection of ROS with various optical probes; and (2) characterization of the porphyrins by the structure–activity relationship (SAR) approach, including determination of the n-octanol/PBS partition coefficients and PS accumulation in Gram-positive and Gram-negative bacteria. Considering that efflux pump inhibitors are promising adjuvant molecules for PDI, we have evaluated the relation between the pump inhibitor verapamil (Ver) and PDI mediated by the investigated photosensitizers. Finally, the effectiveness of neutral, positively, and negatively charged porphyrins in both bacteria cell accumulation and photodynamic activity was compared.

## 2. Results

### 2.1. Photosensitizers

**Figure 1:**
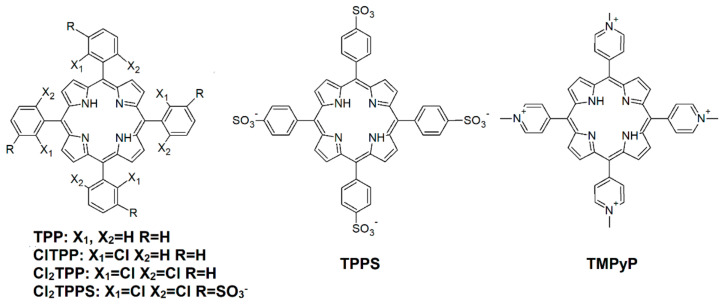
The chemical structures of the investigated porphyrins.

The starting points of our studies are the commercially available porphyrins with different molecular charges, namely, *meso*-tetraphenylporphyrin (TPP), *meso*-tetra(4-sulfonatophenyl)porphyrin (TPPS), and 5,10,15,20-tetrakis(1-methyl-4-pyridinium)porphyrin (TMPyP). We have also examined the photoactivity of some rationally designed porphyrins with various numbers of chlorine atoms in the *ortho* position of the phenyl rings: the chloro-substituted porphyrins, such as 5,10,15,20-tetrakis(2-chloro-phenyl)porphyrin (ClTPP), 5,10,15,20-tetrakis(2,6-dichloro-phenyl)porphyrin (Cl_2_TPP), and 5,10,15,20-tetrakis(2,6-dichloro-3-sulfonatophenyl)porphyrin (Cl_2_TPPS). Their chemical structures are presented in Figure 1. The synthetic procedure of the porphyrins described as ClTPP, Cl_2_TPP and Cl_2_TPPS was previously published [17,19,52,53,54]. The substitution of macrocycles with sulfonic groups provides solubility in water but does not prevent aggregation in a polar solvent such as PBS.

### 2.2. Spectroscopic Characterization of the Photosensitizers

**Figure 2:**
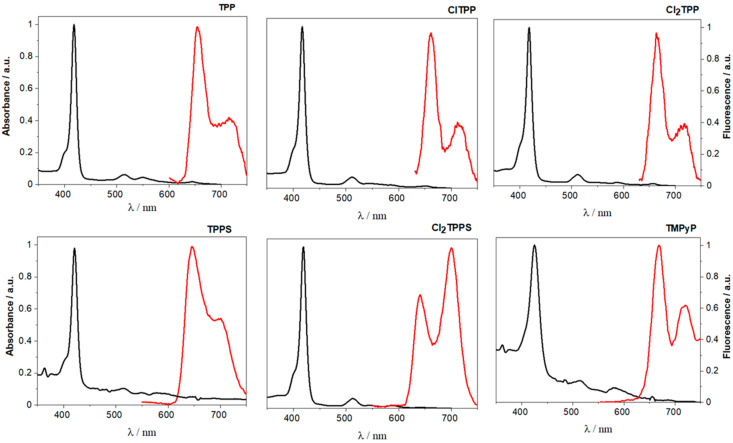
Normalized electronic absorption and emission spectra of the studied porphyrins measured in DMSO solution at room temperature.

The UV-Vis electronic absorption spectra of TPP, ClTPP, Cl_2_TPP, TPPS, Cl_2_TPPS, and TMPyP registered in DMSO at room temperature are presented in Figure 2. The intense Soret band attributed to S_0_→S_2_ transitions is observed at ~420 nm for all the tested compounds. Furthermore, the studied porphyrins possess a characteristic absorption in the 500–700 nm region (Q-bands), which corresponds to the electronic transitions between the S_0_→S_1_ states. While PDT agents in anticancer treatment must strongly absorb above 630 nm, blue light is more proper for treating infectious diseases [55]. The lower optical penetration depth of the blue photons in comparison to the red ones allows for their microbial photoinactivation with negligible keratinocyte damage [56]. The absorption at ~420 nm, due to the high molar absorption coefficients (Table 1 and Figure S1), can be useful in antimicrobial therapy and require relatively low light doses. However, metal-free porphyrins are known to form aggregates in an aqueous solution. The substitution with sulfonate groups modulates the solubility of porphyrin, changes the spectroscopic properties, and may also lead to J-type aggregation, which depends on the concentration and pH value [57]. Therefore, to avoid this undesirable process causing ineffective ROS generation and reducing the biological activity, in these studies, we involved the porphyrin derivatives bearing electron-withdrawing groups. The introduction of chlorine atoms in the *ortho* position of the phenyl rings significantly reduces aggregation and affects the redox properties, which results in the stabilization of the porphyrin structure against (photo)oxidation.

Fluorescence spectra of the tested porphyrins well correspond with their electronic absorption spectra (Figure 2), thus confirming the purity of the samples and lack of aggregation. The fluorescence quantum yield is a measure of the number of photons emitted from the ones that were absorbed (Equation (S1)), whereas the fluorescence lifetime of a species is related to the magnitude of its fluorescence quantum yield. TPP is used as a well-established reference for determination of the photophysical properties of modified porphyrins ($\Phi_{F}$ = 0.10 in toluene) [19] It is observed that substitution with sulfonate groups does not change the fluorescence quantum yield (0.10) and slightly increase the fluorescence lifetime from 10.1 ns (TPP) to 11.5 ns (TPPS). The substitution with 4 chlorine atoms (ClTPP) decreases the quantum yield to 0.04 and shorten the fluorescence lifetime to 7.9 ns. Further substitution with eight chlorine atoms (Cl_2_TPP, Cl_2_TPPS) decreases fluorescence quantum yield even more significantly to 0.02 and changes the τ_F_ to 4.9 and 6.0 ns, respectively. The time-resolved fluorescence profiles of each porphyrin are presented in Figure S2, summarized in Table 1, and are in good agreement with the data reported in the literature [60]. Thus, the rate of ISC to the triplet state should also be enhanced. This effect is further intensified by the heavy atom effect, as previously observed for a series of fluorinated derivatives [61]. The values of the spin-orbit coupling constants (ζ) derived from atoms with higher atomic numbers in the structure are as follows: ζ = 0.24 for H, ζ = 269 for F, and ζ = 586 for Cl. Accordingly, our results show a much shorter $\tau_{F}$ and remarkably lower $\Phi_{F}$ for the chlorinated derivatives. To sum up, chloro-substituted porphyrin derivatives are characterized by enhanced optical properties compared to their unsubstituted counterparts. Modification with chlorin atoms also positively affects the fluorescence lifetimes and singlet oxygen quantum yields. These properties should therefore improve in vitro biological activity, as they reduce the concentration of the porphyrins needed to achieve a similar phototoxicity.

### 2.3. Theoretical Calculations

The theoretical calculations performed for the studied photosensitizers in the ground state confirm that the addition of halogen atoms in the *ortho* position of the phenyl rings leads to the change of geometry (torsion angle) caused by steric repulsion of the bulky groups [62]. The torsion angle (φ) between the porphyrin macrocycle and phenyl group in TPP, TPPS, and TMPyP is about 60° and has changed to ~90° along with the halogen atoms’ substitution (Figure S3). The DFT studies showed more in-plane conformation for tetraphenyl porphyrin than for its halogenated derivatives, leading to a conjugation extension. Moreover, negatively charged TPPS is known to form aggregates, resulting in edge-to-edge stacking, while TMPyP is found to form a dimer in water [63,64]. A change in geometry after substitution with chlorine atoms results in a diminished aggregation by minimizing the electrostatic π−π repulsive interactions. The absorption spectra and singlet-triplet energy gaps were computed using the development of the time-dependent density functional approaches (TDDFT). The computed UV–vis spectra of the studied porphyrins are in good agreement with the experimental spectra (Figure S4). The molecular orbital contribution in the water environment is presented in Table S1 and corresponds to Gouterman’s model. Moreover, the plot of the highest occupied molecular orbital (HOMO) and lowest unoccupied molecular orbital (LUMO) isodensity confirms that the electronic transition occurs between the π and π* orbitals (Figure S5). The blue shift was noticed for halogenated derivatives between the S_0_ and S_1_ transition (Figure S6). The computed value of oscillator strength ca. 1.24–1.80 confirms the strong and high absorption in the UV-Vis range (Table S1). The highest oscillator strength in the Soret band is observed for TPPS (1.80). However, the theoretical calculations (water environment, not PBS) do not correspond to the experimental condition where the Soret band is shifted and has a reduced intensity. Cl_2_TPPS also shows high oscillator strength (1.61), suggesting its highest molar absorption coefficient in the water environment. The transition between the singlet ground state (S_0_) and singlet first (S_1_) or second (S_2_) excited state can lead to a transfer of energy to the excited state with a different multiplicity (T_1_) through an intersystem spin crossing (ISC). The T_1_ energy can be transferred to an oxygen molecule (^3^Σ_g_ ground state) with the same multiplicity, leading to singlet oxygen generation. In the case of porphyrins, the long-lived triplet state has an energy E_T1_ = 33–36 kcal/mol [60], as confirmed in this study for TPP (E = 32.3 kcal/mol) and TMPyP (E = 34.8 kcal/mol), respectively. The energy of the second excited molecular state of oxygen (^1^∆_g_) is E_Σ_ = 37.5 kcal/mol, while the energy of singlet oxygen (^1^Δg) is equal to E_Δ_ = 22.5 kcal/mol [65]. Therefore, this energy is sufficient for a type-II mechanism for all the analyzed photosensitizers (Table 2).

**Figure 3:**
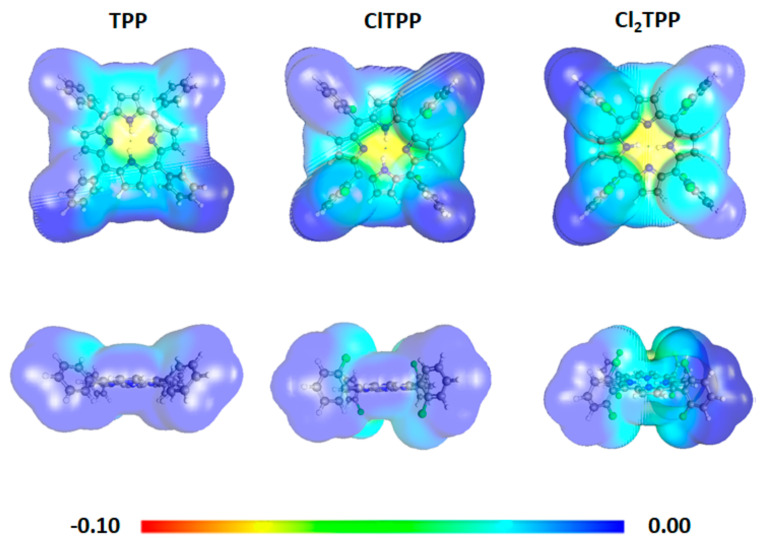

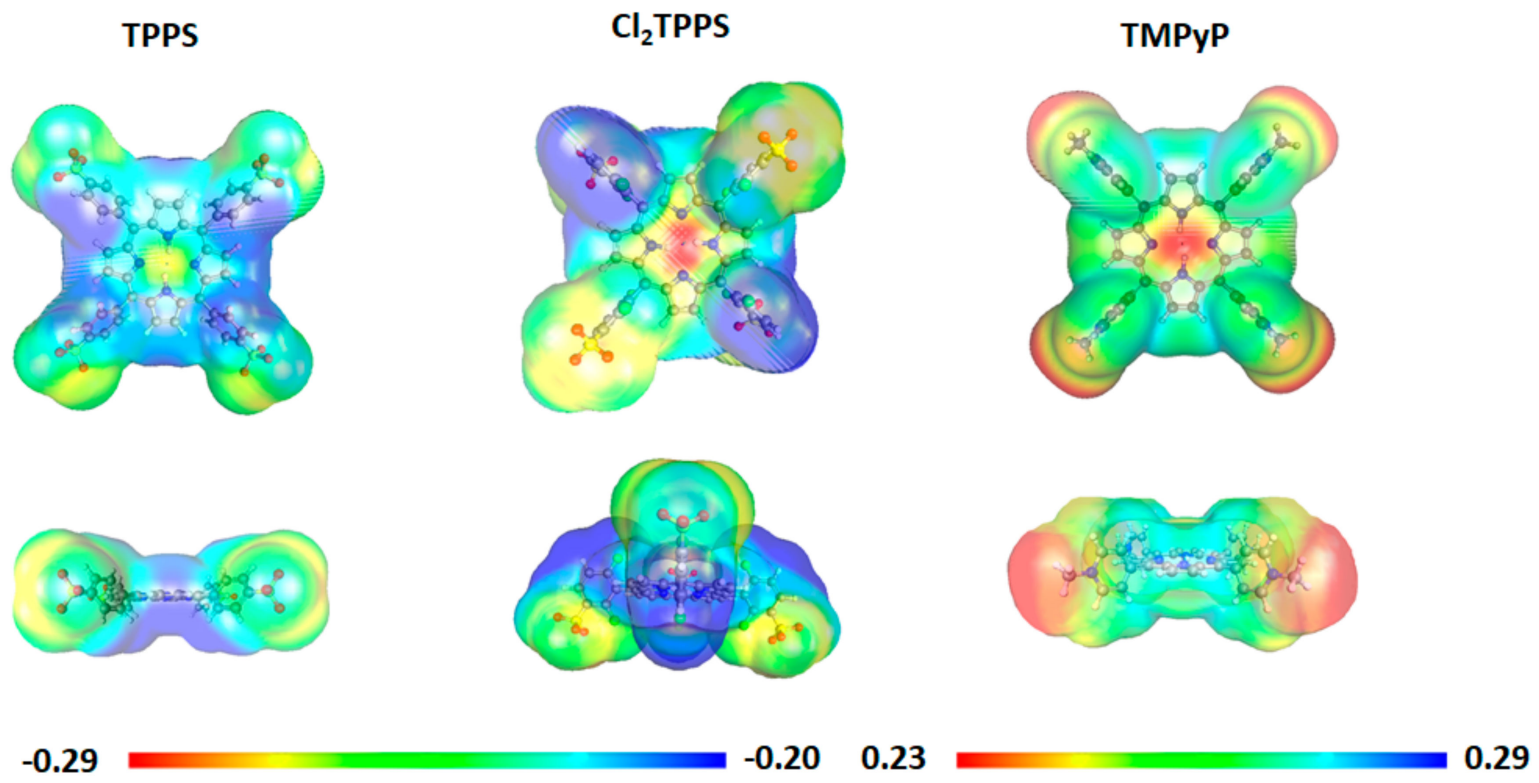
Electronic density maps of the photosensitizers from the total self-consistent field density mapped with the electrostatic potential, isovalue = 0.0004, at the B3LYP/6-31G(d) level in the atomic units.

The important structural factor that influences the accumulation of the photosensitizer in the bacteria cells is a charge distribution in the molecule [66]. The substitution with a chlorine atom in the TPP structure has a minor impact on the charge distribution. However, the DFT calculations at the M06/6-31G(d) level revealed that symmetrical Cl_2_TPP possesses a slightly higher positive charge density around the macrocycle than TPP and ClTPP. The calculation performed for the positively charged porphyrin (TMPyP) showed a significant positive charge density closed to the macrocycle rather than on the peripheral position (Figure 3) [67]. The difference between the synthesized Cl_2_TPPS and commercial TPPS also lies in the location of the -SO_3_H group in the *para* and *meta* positions, respectively (Figure 1). The asymmetric porphyrins with the peripheral charged group have an amphiphilic character, leading to a better accumulation in cells. The *meta* substitution reduces the symmetry and, therefore, the distribution of the charge from negative (TPPS) to both a positive and negative charge density (Cl_2_TPPS) around the molecule (Figure 3). From the computed isodensity maps, it is clear that Cl_2_TPPS shows widespread charged in the molecule and thus has potentially better accumulation in cells.

### 2.4. Cyclic Voltammetry

**Figure 4:**
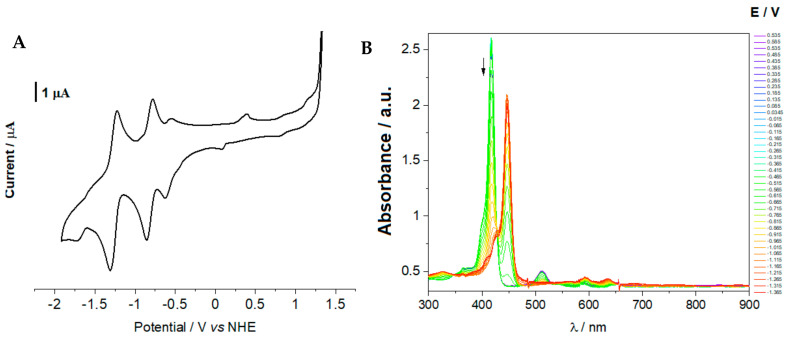
The cyclic voltammograms of ClTPP (concentration 0.5 mM) in DMSO in 0.2 M TBAP 20 mV/s with a vitreous carbon working electrode (**A**), and the absorption spectra of the ClTPP recorded for different applied electrode potentials (**B**).

The redox properties of TPP, ClTPP, Cl_2_TPP, TPPS, Cl_2_TPPS, and TMPyP were studied using cyclic voltammetry. The cyclic voltammograms recorded for the studied molecules are shown in Figure 4A and Figure S6. For comparison, the determined potentials are given vs. NHE and are summarized in Table S2. Particular attention has been paid to the reduction potentials, which can correspond to the excited states. As previously reported, porphyrins exhibited one ring-centered reduction ca. −1.5 V vs. the SCE (the excited state potential) and one or two ring-centered oxidations ca. 0.5 V vs. the SCE (TMPyP) [61,68]. Additionally, the oxidation potentials for the sulfonated derivatives (TPPS and Cl_2_TPPS) versus NHS were determined and found to be 0.22 V and −0.16 V, respectively (Figure S6). Figure 4 shows two reduction values that fall within the range in which the excited state’s potential can be expected. For the remaining porphyrins in the given range, even several potentials values can be found, indicating a reduction in porphyrin. The spectroelectrochemical measurements were performed to correctly determine the one-electron reduction of the ring related to the excited state potential.

**Figure 5:**
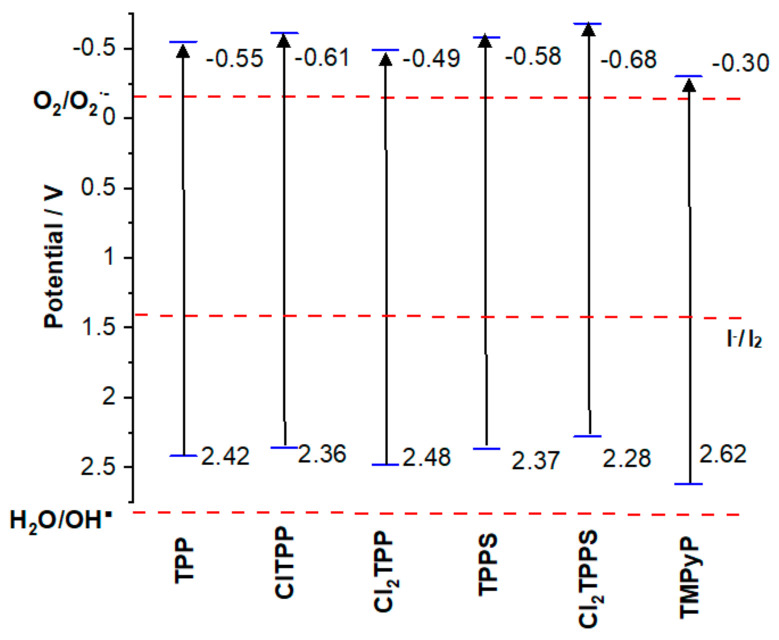
The comparison of the HOMO–LUMO level of porphyrins with water, oxygen, and iodine redox potential. The blue lines represent the potentials determined from the electrochemical measurements and the purple lines indicate values from the photoelectrochemical measurements.

Figure 4B shows the spectral change upon reduction of ClTPP. The addition of one electron to ClTPP (similarly for all studied porphyrins, Figure S7) is accompanied by the loss of the intensity of the Soret band at 420 nm along with the appearance of a new band at 448 nm and a broadband at 600–900 nm, which is typical for porphyrin π-anion radicals [61]. The potential is lower than this value, which is accompanied by the changes in the ClTPP absorption spectrum, coinciding with a reduction at −0.61 V in Figure 4B. The potentials for which we also observed the Soret band’s disappearance are interpreted as the excited-state potentials (Figure 5 and Table S3). Based on the determined LUMO level, the ground state was assigned by adding the Soret band energy. All the studied photosensitizers in the excited states can transfer an electron to the acceptor (O_2_) with O_2_^•−^ generation (type I mechanism) (Figure 5). For this reason, the photodynamic effectiveness depends on the O_2_ concentration from the air or environmental enrichment with molecular oxygen [26]. Hypothetically, compounds with a HOMO level (ground state potential) below 2.80 V after excitation may oxidize H_2_O to a hydroxyl radical with one electron process with ROS generation in the absence of oxygen. The highest LUMO level determined by spectroelectrochemistry was −0.30 ± 5 V for TMPyP. Considering the ∆E gap between HOMO and LUMO is 2.92 eV, the HOMO level is equal to 2.40 V. This is still below the 2.80 V required for hydroxyl radical generation by a one-electron reaction (Figure 5). Thus, the only possible pathway to produce HO^•^ radicals is a three-electron reduction [69].

The addition of inorganic iodine salt in the presence of singlet oxygen may produce highly reactive iodine species, including iodine radicals (Equation (1)) [31]. Moreover, the excited porphyrin may oxidize iodide ions to iodine radicals (Figure 5; Equation (2)). The iodine radical and further iodine may also be generated through a reaction with the hydroxyl radical (Equation (3)).

^1^O_2_ + I^−^ → O_2_^•−^ + I^•^ (1)

2I^−^ − 2e^−^ → I_2_ (2)

2HO^•^ + 2I^−^ → 2HO^−^ + I_2_ (3)

### 2.5. Mechanistic Studies

#### 2.5.1. Singlet Oxygen Quantum Yields

**Figure 6:**
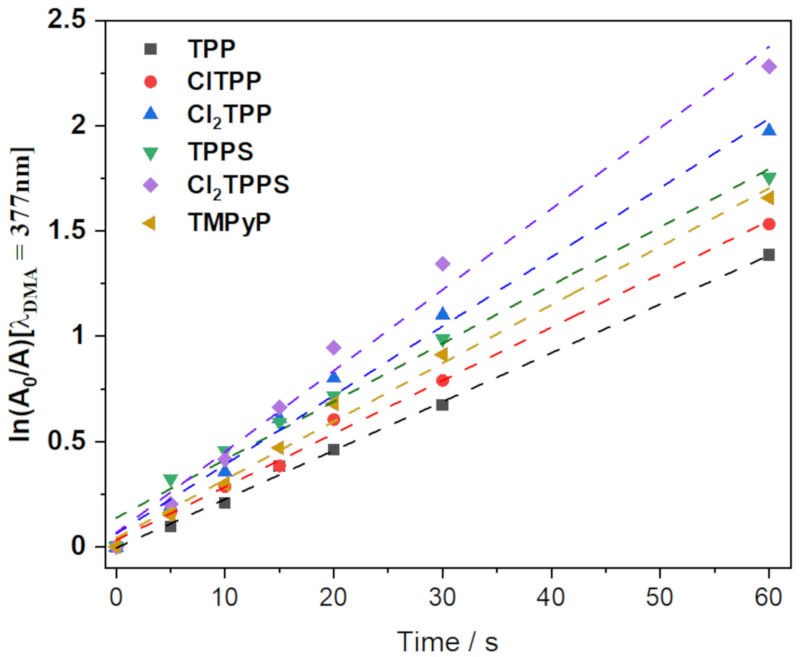
Singlet oxygen quantum yield determination using 9,10-dimethylanthracene (DMA) as a singlet oxygen quencher. There is a decrease in DMA absorption measured in PBS (with 0.5% DMSO) in the presence of porphyrins under the irradiation with visible light using a 420 nm (±20 nm) diode as the light source.

The efficiency of energy transfer from PS in its triplet excited state to ground state molecular oxygen is crucial for the efficient generation of singlet oxygen [16]. The increasing rate of ISC results in an observed rise in singlet oxygen formation. The singlet oxygen quantum yield was evaluated using a chemical quencher—9,10-dimethylanthracene (DMA)—in the presence of each porphyrin solution. The presence of four chlorine atoms in the structure increased the singlet quantum yield (Φ_∆_) from 0.60 (TPP) to 0.65 (ClTPP), while a further substitution with 8 chlorine atoms (Cl_2_TPP) increased Φ_∆_ to 0.85. Analogically, the substitution of TPPS with chlorine atoms significantly increased the singlet oxygen generation (Figure 6, Table 1). The highest quantum yield of singlet oxygen generation was determined for Cl_2_TPPS (Φ_∆_ = 0.95).

#### 2.5.2. Photoactivity of Photosensitizers

**Figure 7:**
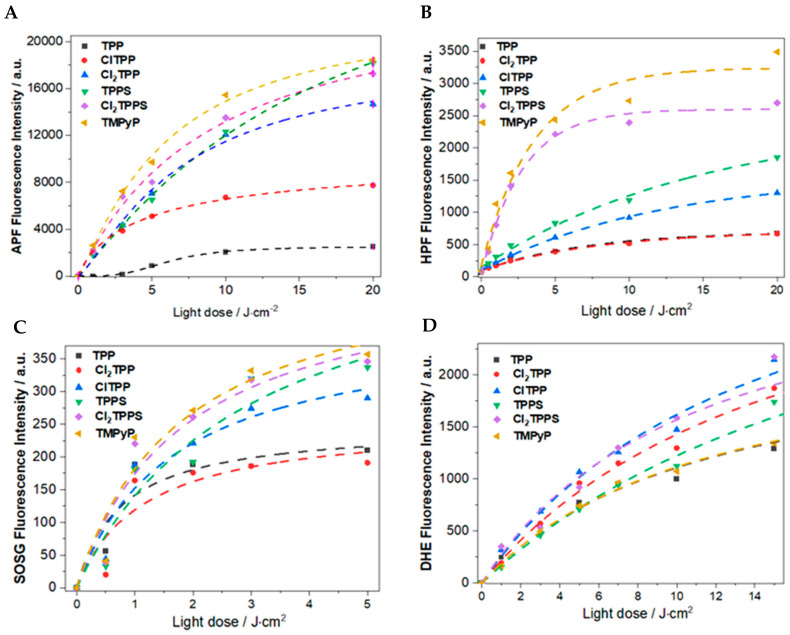
Photogeneration of ROS: the fluorescence intensity from ROS probes (10 µM), namely, APF (**A**), HPF (**B**), SOSG (**C**), and DHE (**D**), during irradiation of a porphyrin solution with a 420 nm LED.

Besides singlet oxygen, other ROS, such as hydroxyl radicals, peroxide anions, and hydrogen peroxide, are important molecules inducing oxidative stress in bacteria. Therefore, singlet oxygen (^1^O_2_), superoxide ions (O_2_^•−^), and hydroxyl radicals (HO^•^) were also detected in the solution of porphyrins in PBS under blue light irradiation. Detection of these ROS was possible by using APF, SOSG, HPF, and DHE fluorescent probes. The high quantum yield of singlet oxygen production by TMPyP and Cl_2_TPPS was further confirmed with experiments involving the SOSG fluorescent probe (Figure 7A). The oxygen reduction to O_2_^•−^ was followed by a reaction with the DHE probe sensitive to superoxide ions and sensitive in the presence of H_2_O_2_ (Figure 7B). Hydroxyl radicals may be generated through superoxide ions and an H_2_O_2_ reduction due to a very low activation energy barrier [69]. Effective HO^•^ generation was observed for the TMPyP, Cl_2_TPPS, and TPPS porphyrins with the APF as a probe for ROS. Additionally, the HPF that is more specific to the hydroxyl radicals shows a significantly higher HO^•^ generation for Cl_2_TPPS and TMPyP (Figure 7D). The results of the photoactivity tests confirm that the presence of chlorine atoms increases the ROS generation capacity. Nevertheless, Cl_2_TPPS indicates superior activity in both type I and type II photogenerated ROS among the chlorinated porphyrin derivatives. Due to the limited stability of these fluorescent probes, the kinetics of the ROS generation has been evaluated in a low-light dose range (up to 20 J/cm^2^).

**Figure 8:**
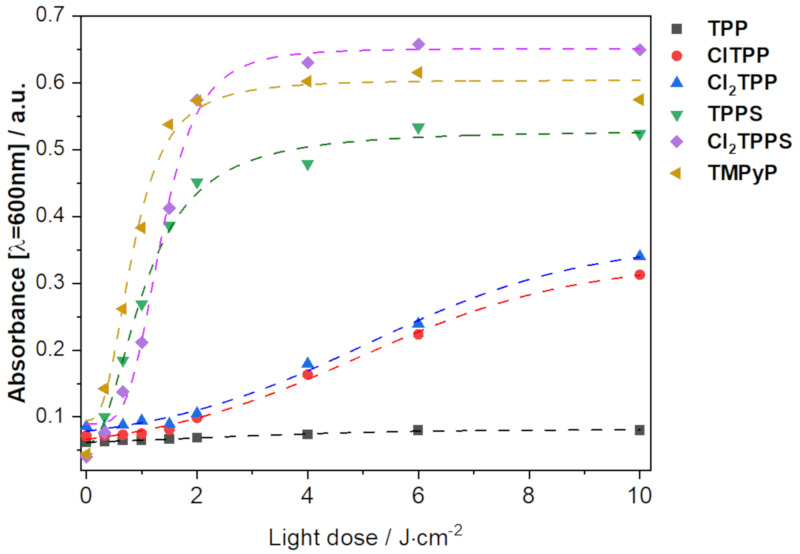
The absorbance of aliquots of photosensitizers (PSs) with 100 mM KI after irradiation with a 420 nm LED with various light doses and added to a starch indicator reagent.

Reactive iodine species, similar to ROS, are characterized by a relatively short lifetime. However, iodine is a stable product that remains even after the PDI procedure. The long-lived iodine species are well known for their antimicrobial properties. Iodine may be detected by the absorbance of its complex with starch (Figure 8). Iodine generation occurs according to the generation of singlet oxygen (Figure 7C, Equation (1)) and hydroxyl radicals (Figure 7A,C; Equation (3)). As a result, iodine is produced by both types of ROS. The addition of KI during the PDI procedure may be especially beneficial for Cl_2_TPPS, TMPyP, and TPPS. However, among all the studied porphyrins, Cl_2_TPPS is the most active in iodine generation (Figure 8). This can be explained by its ability to generate both singlet oxygen and hydroxyl radicals with high quantum yield.

### 2.6. Photostability of the Photosensitizers

**Figure 9:**
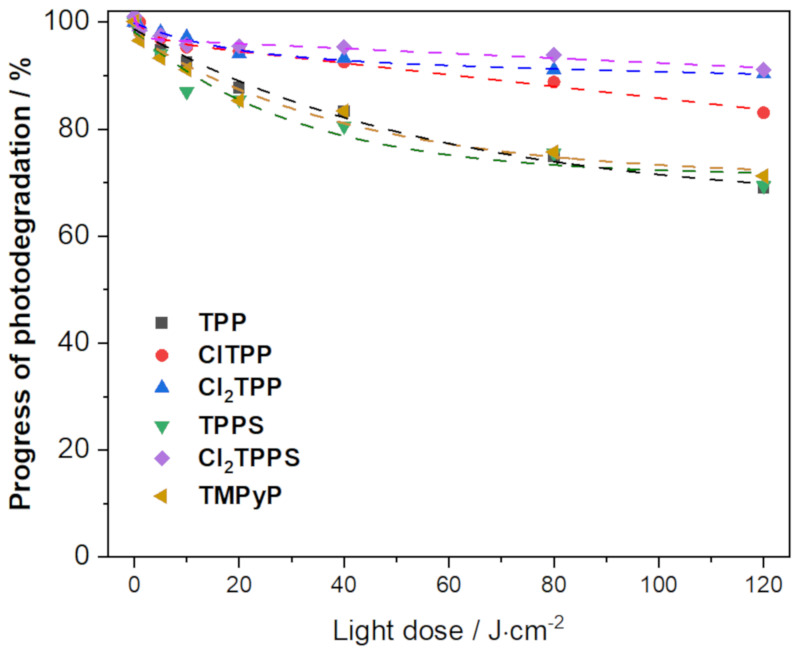
The photostability of porphyrins: TPP, ClTPP, Cl_2_TPP, TPPS, Cl_2_TPPS, and TMPyP in PBS solution (DMSO content < 1%) with an absorbance ca. 1. Irradiation of the porphyrin solution was carried out using a 420 nm LED with various light doses.

An undesirable and frequently occurring phenomenon is the photodegradation of compounds as the results of self-generating ROS under light irradiation [29]. Photosensitizers with lower energy of HOMO are characterized by higher photostability [39]. They are more disfavored to the photooxidation and photo-transformation processes. The theoretical calculations reveal that the chloro-substituted porphyrin derivatives are characterized by lower energy of the HOMOs (TPP > ClTPP > Cl_2_TPP; TPPS > Cl_2_TPPS, Figure S5). The electron-withdrawing effect is responsible for making the reduction easier and oxidation harder. Thus, the progress of the photodegradation depends on the presence of halogen atoms in the phenyl ring’s meta position. The Cl_2_TPPS showed the highest photostability (ca. 93%) after illumination with a total light dose of 120 J/cm^2^ (Figure 9). For comparison, the sulfonic derivatives without a chlorine atom in the structure are significantly less stable (ca. 70%). The same trend was observed when comparing TPP with their halogenated derivatives in both irradiation conditions: 420 nm LED (Figure 9) and xenon lamp (Figure S8). In the case of TMPyP, the energy of HOMO is identical to Cl_2_TPP, although the stability of this molecule is similar to non-chlorine derivatives. However, it is important to note that the final level of degradation also depends on the compounds’ structure and ROS amount generated, which is the highest for TMPyP. Chlorinated derivatives, especially Cl_2_TPPS, exhibit a lower tendency to photodegradation, which ensures that the ROS level is maintained even after a long time of irradiation.

### 2.7. Determination of Partition Coefficients (LogP)

The balance between the hydrophilic and hydrophobic moieties and the lipophilicity of the PS has been shown to have a strong influence on the PDT/PDI efficacy. The n-octanol/water partition coefficient (logP) is well established to predict the biological activity of drugs and other biologically active compounds. The results presented in Table 3 indicated that the most hydrophobic photosensitizer is an unsymmetrical mono-substituted derivative (ClTPP). The presence of a negatively or positively charged group significantly enhanced its solubility in an aqueous environment. The substitution of a hydrophobic TPP with tetra(4-sulfonatophenyl) or tetra(1-methyl-4-pyridinium) groups significantly changes the physicochemical properties of the molecules, making them more amphiphilic. The sulfonic derivatives are the most hydrophilic. The functionalization of TPPS with the chlorine atoms (Cl_2_TPPS) slightly decreased the solubility in aqueous media.

While sulfonated derivatives (TPPS and Cl_2_TPPS) are hydrophilic with a negative logP value, TMPyP, Cl_2_TPP, ClTPP, and TPP are more lipophilic with progressively higher positive logP values. The cell membrane of a mammalian cell comprises double-layered phospholipids, and hydrophilic molecules cannot permeate the cell membrane, while amphiphilic and hydrophobic molecules can easily permeate the cell membrane and be promptly transported into the cell. However, bacteria also have a cell membrane composed of double-layered phospholipids but encased in a cell wall. More hydrophilic photosensitizers are likely relatively better at inactivating bacteria. Perhaps, they can at least bind to the cell wall, if not penetrate the cell membrane. More hydrophobic photosensitizers can penetrate the cell membrane of the much bigger mammalian cells, but bacteria’s cell walls mostly exclude them.

### 2.8. The Accumulation of Photosensitizers into Bacteria Cells

**Figure 10:**
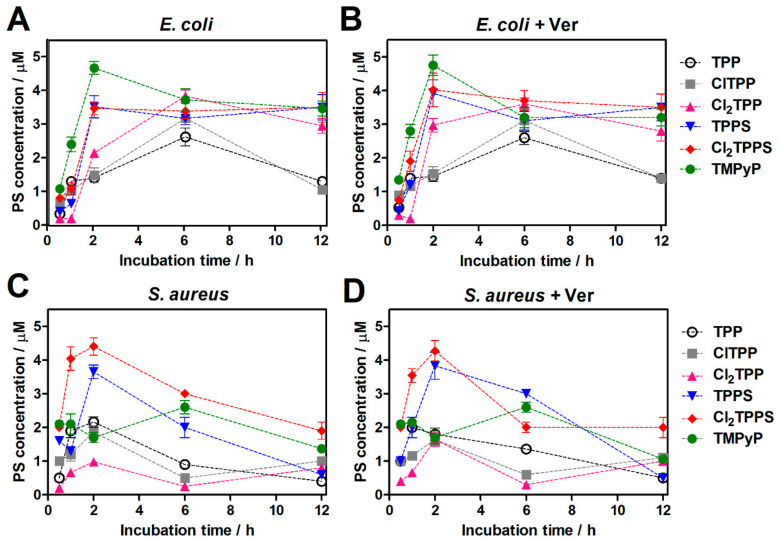
Time-dependent cellular uptake of PS with the initial 20 μM concentration by *E. coli* without (**A**) and with verapamil (**B**) and *S. aureus* without (**C**) and with verapamil (**D**), for various incubation times.

We assessed the uptake of cationic (TMPyP), neutral (TPP, ClTPP), and anionic photosensitizers (TPPS, Cl_2_TPPS) by Gram-positive (*S. aureus*) and Gram-negative (*E. coli*) bacteria. The optimal time of porphyrin accumulation was determined by incubation of the bacteria suspensions with photosensitizer solutions (20 μM) ranging from 0.5 h to 12 h. The PS concentration was determined in the bacteria cells after being lysed in SDS 10% (Figure 10). The results showed the degree of accumulation of the investigated porphyrins. The optimal time for PS incubation with bacteria (*E. coli*, *S. aureus*) was 2 h and, after that time, there is no higher PS concentration in the bacteria cells.

**Figure 11:**
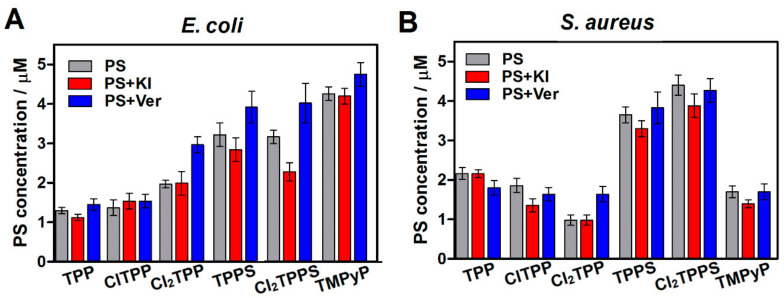
Uptake of PSs (with the initial 20 μM concentration) by *E. coli* (**A**) and *S. aureus* (**B**) with verapamil and KI addition.

The possibility of using shorter incubation times in PDI than for typical photodynamic effect against cancer cells results from the fast-electrostatic interaction between the cationic functional groups in the PS structure and the negatively charged teichuronic and lipoteichoic acids located in the outer wall of bacterial and fungal cells. The prolongation of incubation time (from 2 h to 12 h) brings no significant increase in cell-bound PS. This feature plays a crucial role in determining the antimicrobial cells’ selectivity toward the host eukaryotic cells. However, the electrostatic interaction between the PS with the charged sites at the bacteria results in binding the cationic molecules to the lipopolysaccharides’ (LPS) outer surface [29,70]. The positive charge of TMPyP led to significantly higher uptake in *E. coli*, as described previously [67,71,72,73]. We have shown that despite the high activity of efflux pumps in the Gram-negative bacteria, the long-term accumulation up to 6 h–12 h (especially for non-charged porphyrins) of PSs increases their affinity to bacteria cells. It most probably demonstrates sticking to the outer membrane by the PS. For Gram-positive bacteria, the uptake of sulfonic porphyrins (TPPS, Cl_2_TPPS) is higher than in Gram-negative species (Figure 11). The comparison between PS concentrations with and without verapamil addition suggests that the efflux pump blockade may increase the PS accumulation, especially in *E. coli*. In the case of *S. aureus*, reduced accumulation of PS was observed after 6 and 12 h of incubation, which may be related to the addition of verapamil and cationic dye extrusion as potential substrates of MEP [50,74]. The highest accumulation in *S. aureus* was observed for negatively charged TPPS and Cl_2_TPPS [75]. In this case, there is no significant effect on the addition of the efflux pump inhibitor—verapamil. In the case of *E. coli*, the concentration of the PS does not change after 2 h of incubation or even slightly increases, which may confirm that the PS is attached to the bacterial cell wall (Figure 11). However, verapamil also influences porphyrin penetration into Gram-negative bacteria at shorter incubation times. To exclude the effect of increasing the external permeability of the membrane of the Gram-negative bacteria with the addition of KI salt, we also checked the accumulation with these monovalent cations. The divalent metal cations Ca^2+^ and Mg^2+^ increased, displacing the native cations from the membrane and enhancing the permeability of the outer membrane [67,76]. The impact of monovalent cations (K^+^) on bacteria cell wall permeability was not observed, even a slight decrease in PS accumulation was noticed (Figure 11).

No significant differences in the accumulation of neutral porphyrins were observed. Results from the bacterial lysates show that Cl_2_TPP is slightly better attached to *E. coli* than the non- and monochloride derivatives, while ClTPP shows higher accumulation in *S. aureus*. On the contrary, in Gram-positive bacteria, a higher accumulation was observed for TPP than for the chlorinated derivatives, but this change is rather insignificant.

**Figure 12:**
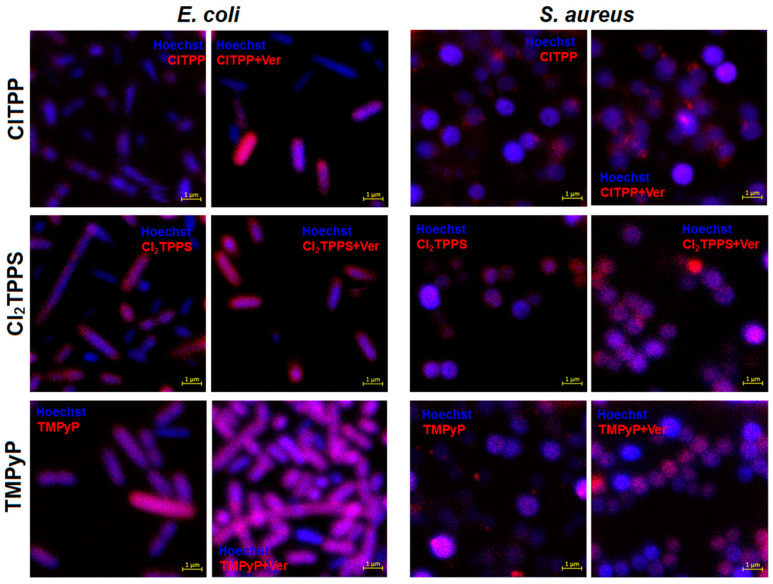
Laser scanning confocal microscopy imaging of porphyrin accumulation with different overall charges (ClTPP, Cl_2_TPPS, and TMPyP) and with the addition of verapamil (Ver) in *E. coli* and *S. aureus*.

**Figure 13:**
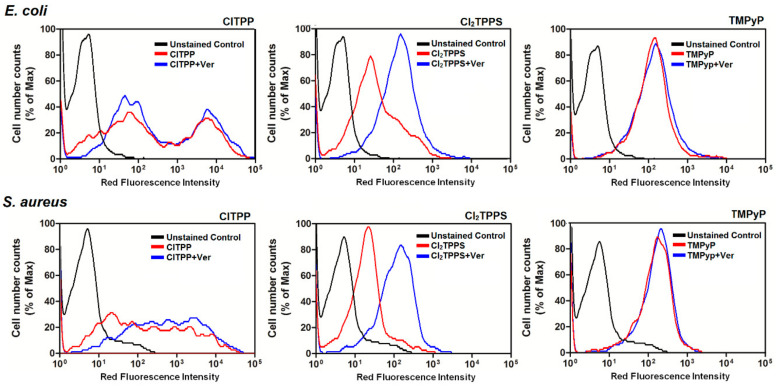
Cellular uptake was determined in *E. coli* (upper) and *S. aureus* (lower panel) based on the red fluorescence of the selected porphyrin (ClTPP, Cl_2_TPPS, and TMPyP) using flow cytometry. Histograms of the fluorescence intensities of the unstained control (black line), a photosensitizer (red), and PS uptake after the addition of verapamil (blue).

For further uptake studies, we investigated and confirmed PS accumulation with confocal microscopy (Figure 12) and flow cytometry (Figure 13). For this reason, we selected the three most representative photosensitizers with a different molecular charge (ClTPP, Cl_2_TPPS, and TMPyP) due to its most prominent uptake in each studied microorganism. The LSCM data are complementary to the uptake studies performed based on the cellular extracts. The LSCM confirms the higher accumulation of PSs after efflux pump inhibition by adding verapamil (100 μM) in *E. coli* but not precisely for *S. aureus* (Figure 12).

**Figure 14:**
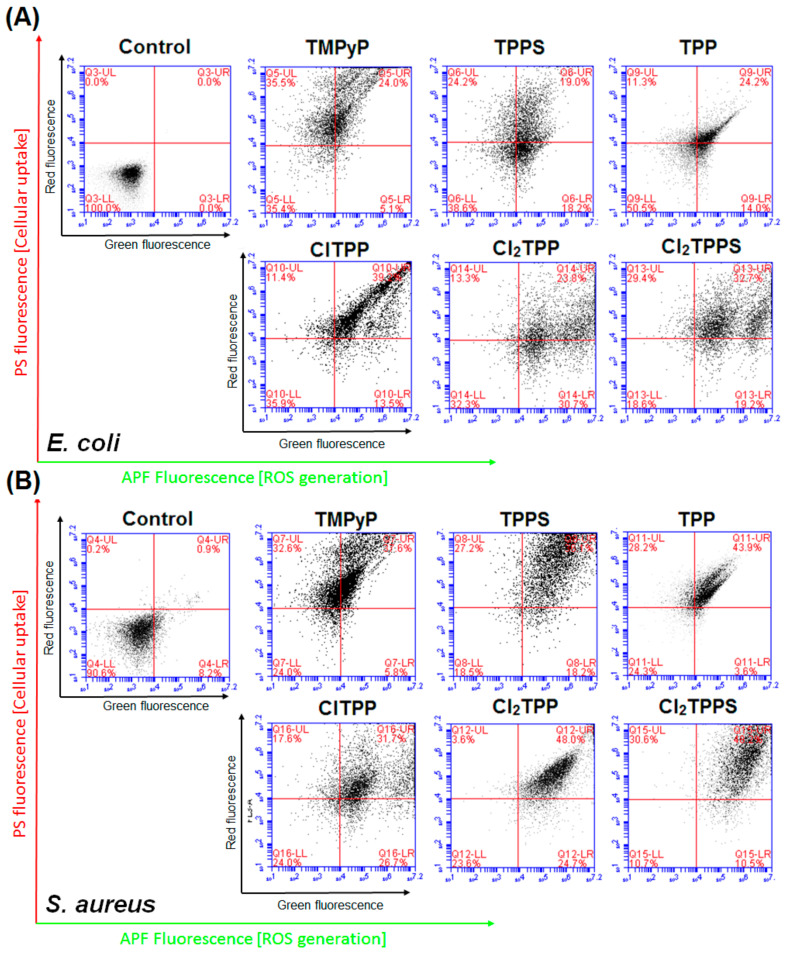
Flow cytometry analysis of reactive oxygen species (ROS) generation *in vitro* in (**A**) *E. coli* and (**B**) *S. aureus*. After incubation of bacteria with PS (2 h) and APF (2 h), the cells were irradiated with a sublethal light dose 10 J/cm^2^. The APF fluorescence signal monitored the level of ROS in the green channel, and the cellular uptake (red porphyrin fluorescence) was detected as an increased red fluorescence signal.

The ability of the tested porphyrin to form ROS in vitro was evaluated with flow cytometry. Using the total cellular fluorescence as a measure of ROS formation, the results indicate the amount of ROS for each photosensitizer accumulated in the bacteria under biological conditions. For this purpose, *E. coli* and *S. aureus* was incubated with the APF probe (25 µM) and each porphyrin (20 µM) for 2 h. Moreover, since SOSG does not pass the bacteria cells, we used APF that may be used to examine the red fluorophores in a two-color analysis. The APF-positive population (green fluorescence) in the control may be related to the presence of naturally occurring intracellular pigments (red fluorescence), ROS in bacteria, or APF autofluorescence (Figure 14). However, after incubation with the photosensitizers and exposed to blue light with a light dose of 10 J/cm^2^, the fluorescence signal drastically increased and confirmed the ROS generation by the PSs accumulated in the bacteria cells.

Red fluorescence confirmed the accumulation of PSs in bacteria and correlates with the uptake determined in the bacterial extracts. We indicated the highest accumulation of TMPyP and Cl_2_TPPS in *E. coli* (Figure 11A), and TPPS and Cl_2_TPPS in *S. aureus*, respectively (Figure 11B). Moreover, ClTPP has a better accumulation profile in *E. coli* than symmetrical Cl_2_TPP and generates ROS more efficiently. It is suggested that asymmetrical substitution with a halogen atom is a good approach for designing a PS against Gram-negative bacteria. A correlation between the generation of hydroxyl radicals in bacteria and PS solution was observed, and Cl_2_TPPS was the most active in this aspect (see Figure 7A and Figure 14). However, there is an exception in TMPyP, which effectively generates HO^•^ in solution but accumulates poorly in *S. aureus* and does not generate hydroxyl radicals effectively *in vitro*. Inside *S. aureus* cells, the production of ROS is more efficient than in *E. coli*. It may be related to the intrinsic catalase enzyme (CAT) in *E. coli*, which transforms hydrogen peroxide into water and molecular oxygen [37,77]. This mechanism is especially noticed for PDI with Cl_2_TPP and Cl_2_TPPS. In the case of *E. coli*, some populations of bacteria successfully deactivate ROS (less green fluorescence signals). Nevertheless, there is still a population of cells where the APF signal is very intense. Unlike *E. coli*, no similar effect was observed in *S. aureus*, where photogeneration of the hydroxyl radicals by Cl_2_TPPS occurs very efficiently.

### 2.9. Photodynamic Inactivation of Microorganisms

**Figure 15:**
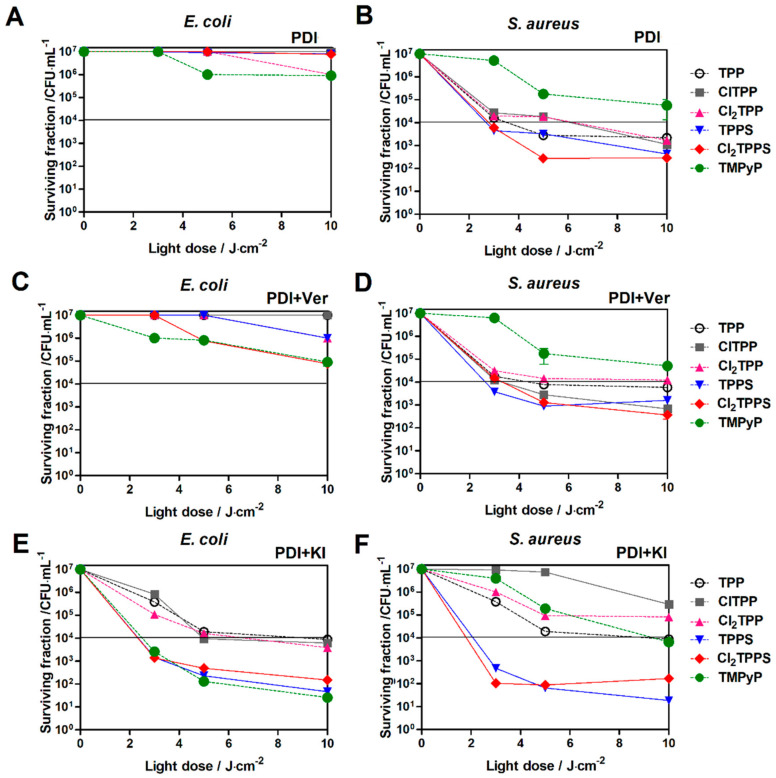
The surviving fraction of bacteria following PDI of *E. coli* and *S. aureus* with TPP, ClTPP, Cl_2_TPP, TPPS, Cl_2_TPPS, and TMPyP as the PS without (**A**,**D**) and with EPI (**B**,**E**) and KI addition (**C**,**F**) after 2 h of incubation with a low light dose up to 10 J/cm^2^.

The evaluation of the photodynamic inactivation of *E. coli* and *S. aureus* using different porphyrin derivatives as photosensitizers (with and without an efflux pump inhibitor (EPI)) was performed to assess the correlation between the molecular charge, cellular uptake, and their ability to disrupt bacteria (Figure 15). The role of a specific microbial EPI, verapamil hydrochloride (Ver), was also investigated in the porphyrin-mediated PDI. Figure 15 shows the surviving bacteria following PDI of *E. coli* and *S. aureus*, respectively, using TPP, TPPS, ClTPP, Cl_2_TPPS, and TMPyP as the PS without (Figure 15A,D) and with an EPI (Figure 15B,E) as well as KI (Figure 15C,F) addition. The survival fraction at 0 J/cm^2^ represents toxicity in the darkness (no dark cytotoxicity). Moreover, there was no significant bacterial viability reduction after exposure to light alone (Figure S9). When combined with light irradiation, all PSs produced a light dose-dependent killing of both types of bacteria, relative to the untreated controls.

**Figure 16:**
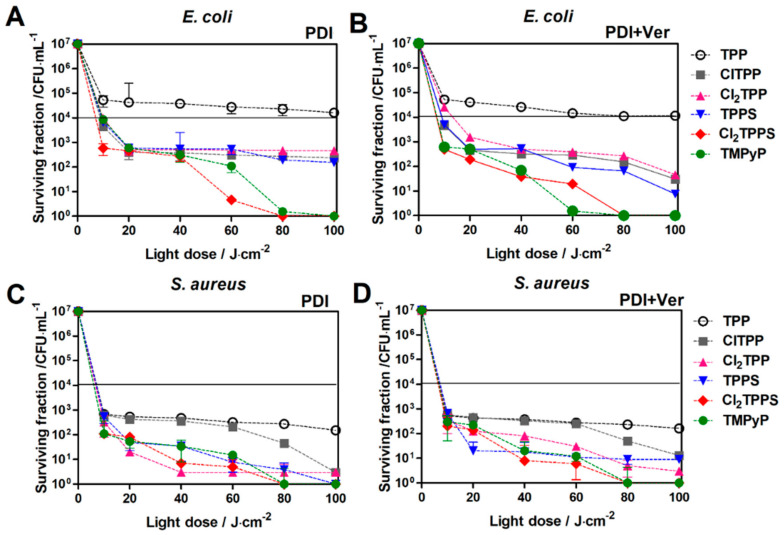
The surviving fraction of bacteria following PDI of *E. coli* and *S. aureus* with TPP, ClTPP, Cl_2_TPP, TPPS, Cl_2_TPPS, and TMPyP as the PS without (**A**,**C**) and with EPI (**B**,**D**) after 2 h of incubation and application of light doses up to 100 J/cm^2^.

**Figure 17:**
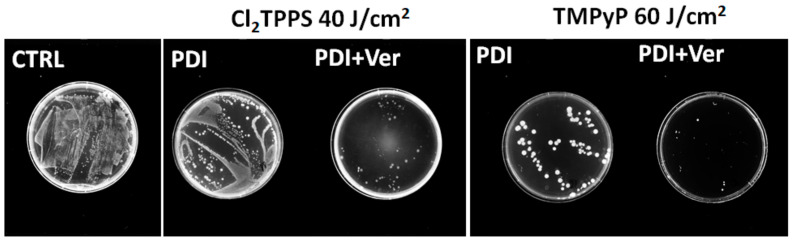
The effect of the EPI on high-dose PDI. The colonies of *E. coli* grew on agar plates after PDI mediated by a Cl_2_TPPS of 40 J/cm^2^ and TMPyP of 60 J/cm^2^, with and without verapamil addition.

The photoactivity against microorganisms shows a strong correlation with PS uptake (see Figure 11). As an example, the PDI with Cl_2_TPPS as a photosensitizer led to the 5 logs of *S. aureus* killing even with a low light dose (5 J/cm^2^). More resistant *E. coli* is hard to deactivate with the standard PDI protocol. Nevertheless, positively charged TMPyP demonstrates the highest photoactivity against *E. coli* at a low light dose (5 J/cm^2^), leading to 2 logs of killing (Figure 15B). Therefore, the inhibition of efflux pumps was used to enhance the PDI effect by increasing the PS’s accumulation in the bacteria cells. Verapamil provided an enhanced photodynamic effect (1–2 logs) against *E. coli* with TMPyP, TPPS, and Cl_2_TPPS (Figure 15C,D). This effect suggests that the PDI is mediated mainly by PS molecules attached to the *E. coli* cell wall and outer membrane via electrostatic interactions between the negatively charged bacteria surface (*E. coli*) and positively charged TMPyP. The applied conditions did not lead to a satisfactory effect of 3 logs killing. Therefore, we decided to use the protocol with the addition of non-toxic iodide salt. The PDI potentiation by the addition of KI corresponds to the starch photo-activity tests (Figure 8), where the highest activity was found for TMPyP, TPPS, and Cl_2_TPPS. The high efficiency of generating reactive iodine species led to a PDI enhancement by 2–3 logs for *S. aureus* and by 5 logs for *E. coli* (Figure 15E,F). However, the PDI effect may be intensified while the PSs are still able to produce ROS prior to possible photobleaching. For this purpose, the PDI was extended to higher light doses up to 100 J/cm^2^ (Figure 16). The complete destruction of Gram-positive bacteria was observed for PDI with a light dose of 40 J/cm^2^, while the Gram-negative bacteria require a light dose of 60 J/cm^2^ to achieve a similar effect. The extended time of the PDI procedure correlates with PS photobleaching. The more photostable the PS is, the higher the light dose (and longer irradiation time) can be applied. In the case of *E. coli*, the most active PS was (as supposed) TMPyP with 7 logs of killing at 80 J/cm^2^ and at 60 J/cm^2^ for the protocol with verapamil. However, Figure 16A,B shows that the PDI of *E. coli* with the sulfonated derivatives (TPPS, Cl_2_TPPS) produced roughly four logs of reduction at 20 J/cm^2^ and seven logs of reduction at 60 J/cm^2^. To visualize the EPI’s influence on high-dose PDI, representative photographs of the agar plates with colonies of *E. coli* treated with PDI and PDI+Ver are shown in Figure 17 (see also Figures S10–S21). A higher activity of Cl_2_TPPS than its non-chlorinated derivatives TPPS was observed (Figure 16A) and corresponds to the quantum yield of singlet oxygen generation (see Figure 6). A PDI with these photosensitizers + EPI resulted in almost the same degree of bacterial killing. These experiments demonstrated the advantage of using sulfonated photosensitizers, especially photostable Cl_2_TPPS, to inactivate Gram-positive bacteria, and are in a good agreement with data published for other anionic photosensitizers (Figure 16C,D) [78].

**Figure 18:**
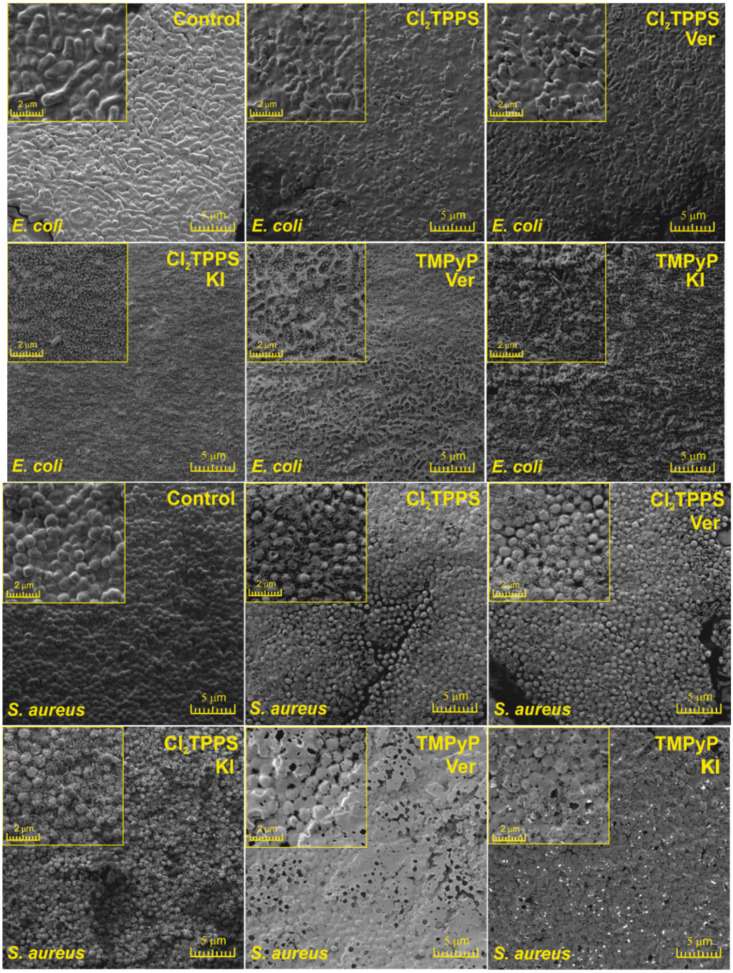
Scanning electron microscopy images of the control and after the PDI-treated bacteria *E*. *coli* (upper part) and *S. aureus* (lower part) with and without the addition of verapamil (Ver) and potassium iodide (KI) at a 10 J/cm^2^ light dose.

**Figure 19:**
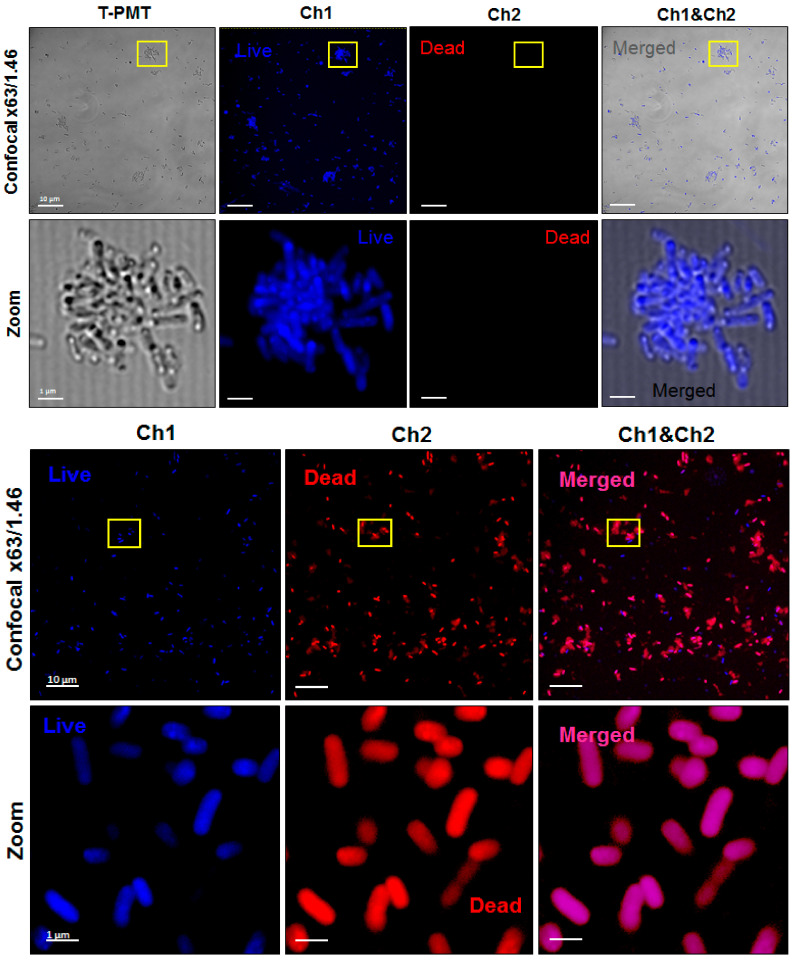

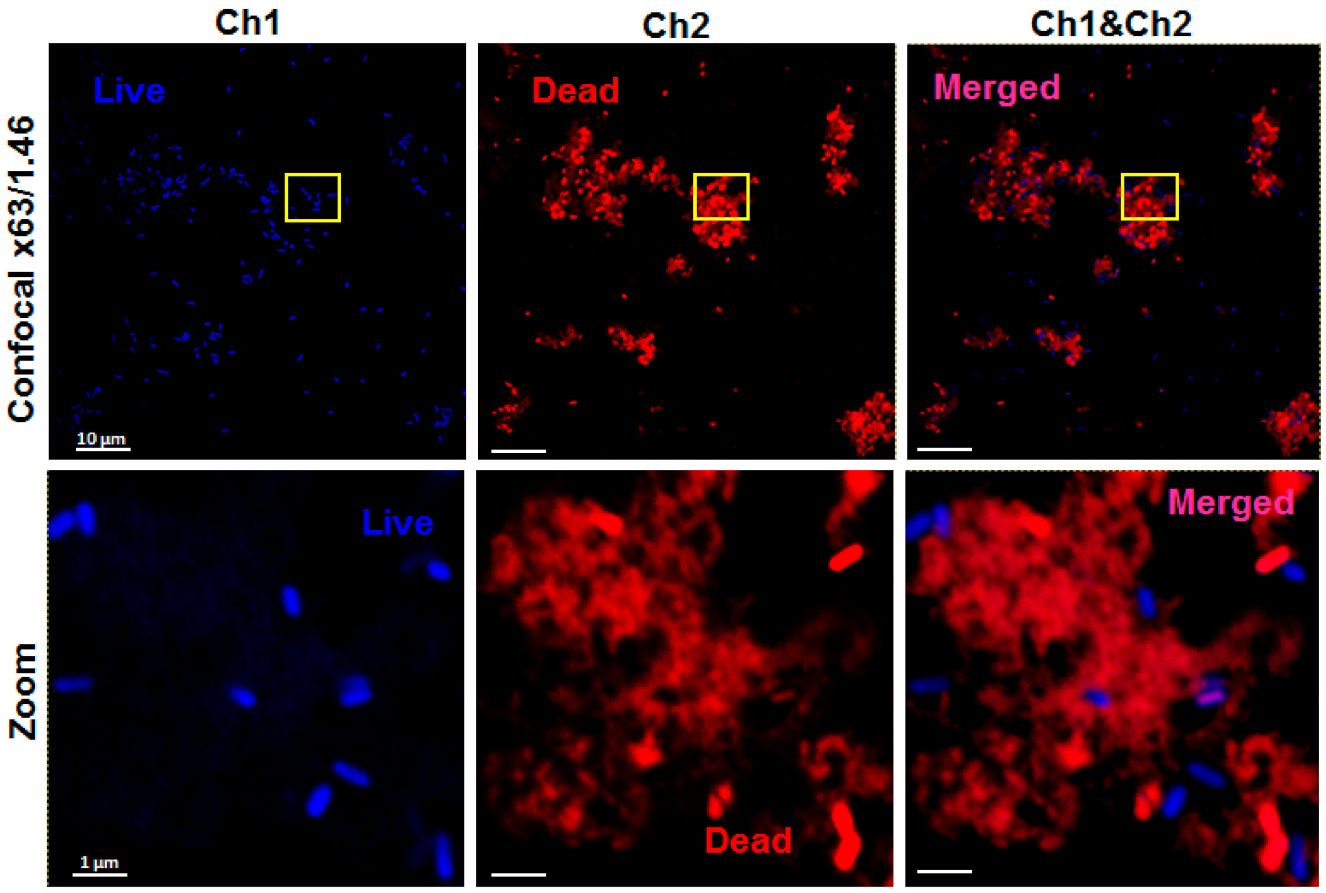
Laser scanning confocal microscopy images of the non-treated (upper panels), PDI-treated (middle), and PDI+KI-treated (bottom panels) *E. coli* stained with Hoechst3342 and PI. The yellows squares on the top panels indicate the zoom area displayed below.

To further visualize the photoinduced damages and the differences in bacterial death mechanisms, we performed SEM imaging immediately after each PDI treatment (PDI alone, PDI in combination with Ver, and with KI, separately). The SEM images of *E. coli* and *S. aureus* are shown in Figure 18. As may be observed, the PDI and PDI+Ver (incubation time: 2 h, 20 µM of PS, and light dose of 10 J/cm^2^) indicated a disruption of bacterial cells and may inhibit the bacteria division without visible membrane disruption. Nevertheless, it is believed that these bacteria were dying or losing cell functions with the intact cell walls (see confocal pictures below, Figure 19). In contrast, after the addition of KI, the cells showed a substantially disrupted cell membrane and even more severe damage.

In further studies, confocal imaging was carried out to confirm the PDI-induced damages in the bacterial cells and distinguish the cellular disruption mechanisms by generating reactive oxygen and iodine species. Figure 19 shows *E. coli* incubated with Cl_2_TPPS for 2 h and irradiated with 10 J/cm^2^ blue light. The imaging was performed immediately after PDI. The stained lived bacteria fluorescence is blue (Hoechst33342), and the disrupted (death) cells were presented in red fluorescence (propidium iodide). The basic PDI protocol can induce moderate photodamages in *E. coli* (blue fluorescence colocalize with red signal), which are the most prominent in cell wall structures. In addition, PDI with KI addition results in complete disruption of the cellular structures and characteristic shapes.

### 2.10. MDR Activity

The effect of PDI with hydrophilic and amphiphilic photosensitizers have a more significant impact on cellular uptake. Interestingly, the light-mediated killing in the presence of verapamil was intermediate between that observed for PDI alone and PDI+KI. There was a remarkable potentiation of PS accumulation in the presence of the EPI, and it appeared that this effect was somewhat more significant for the *E. coli* than for *S. aureus* (Figure 11 and Figure 12). It may suggest that the investigated *E. coli* strain is characterized by higher efflux pumps and MDR activity.

**Figure 20:**
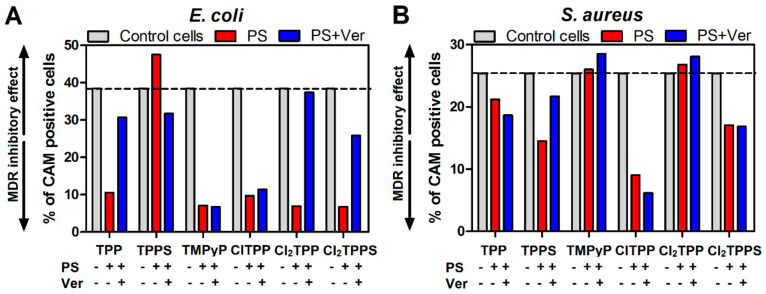
Regulation of drug efflux in the photosensitizer-treated bacteria: (**A**) *E. coli* and (**B**) *S. aureus*. After the treatment of cells with 20 µM PS for 2 h, 0.25 µM calcein AM was added to the cells, and the calcein AM retention was examined. Greater numbers suggest a more significant inhibitory effect on MDR activity.

The activity of the efflux pumps by P-gp-dependent resistance, which may be inhibited by non-toxic agents such as verapamil—a calcium channel blocker—has been checked. In the applied test, a fluorogenic dye—calcein acetoxymethyl ester (calcein AM)—was used. Calcein AM is a non-fluorescent, highly lipophilic soluble dye that can readily cross normal cells’ membranes. Inside the cells, ester bonds are cleaved by endogenous esterases, converting calcein AM to hydrophilic and intensively fluorescent calcein. Calcein is well preserved in the cytosol, and its fluorescence is not pH or calcium dependent. MDR cells have a high abundance of Pgp and rapidly pump non-fluorescent AM calcein out of the membranes, reducing fluorescent calcein accumulation in the cytosol. Their MDR activity was studied in *E. coli* and *S. aureus* cells to clarify whether verapamil indeed regulates photosensitizer efflux. MDR inhibition was examined by measuring calcein AM retention in cells treated with PS alone and treated with PS+Ver. As shown in Figure 20, verapamil addition enhanced the retention of calcein AM in *E. coli*, indicating significant inhibitory effects on MDR activity. Conversely, calcein AM was not significantly altered in *S. aureus*, even with verapamil treatment. The results indicated verapamil-mediated PDI resistance by controlling the efflux of drugs from the bacteria.

The PDI and EPI combination maximized the antibacterial effect, increased the bacteria disruption by ROS, and increased the PS accumulation. Furthermore, ROS generated by PS under blue light irradiation inside bacteria damages the intracellular molecules, including membrane proteins or lipids. According to previous studies, ROS increases non-selectively attack membrane proteins, including efflux pumps [50]. In this process, the proteins necessary for the survival of bacteria are also damaged or suppressed by ROS generation. As a result, ATP cannot be synthesized by ATP synthase (located in the membrane with the bulky hydrophilic catalytic F1 portion sticking into the cytoplasm) and the efflux pumps become dysfunctional due to the lack of energy supply. Therefore, we confirmed the effect of PS retention based on the assumption that ROS will affect cell cytotoxicity and efflux pumps (Figure 20), a characteristic of multidrug resistance. When verapamil inhibited P-gp, the PSs remained in the cytoplasm; a similar pattern was observed during the photodynamic effect. Moreover, the efflux pump activity was observed mainly in *E. coli*, so the targets of resistant *E. coli* may be external structures, such as cell walls and cytoplasmic membranes instead of DNA. This result suggests that ROS production inhibited the drug efflux, thereby further synergizing the photosensitizing effect of the investigated porphyrin derivatives.

## 3. Discussion

Our results presented in this paper confirmed the drawbacks of non-substituted porphyrins (e.g., TPP) related to their insolubility in water and the lack of a charge. Thus, we investigated the structurally modified porphyrins and determined that the chloro-substituted porphyrins (ClTPP, Cl_2_TPP) exhibited improved photophysical properties due to the heavy atom effect, but still are too hydrophobic. Sulfonic acid derivatives bearing a negative charge (TPPS and Cl_2_TPPS) are highly water-soluble and efficiently accumulate in Gram-positive bacteria. However, TPPS suffers from low photostability under high light doses and may be resolved by chlorine atom substitution in the porphyrin structure. On the other hand, a positively charged photosensitizer (TMPyP) is essential for good electrostatic adherence to or penetration through the cell walls of Gram-negative pathogens. Nevertheless, fast photodecomposition did not allow for using the protocol with a long irradiation time (higher light doses). Therefore, we introduced both modifications (halogen atoms and sulfonic groups substituents) to develop photosensitizers characterized by a very high ^1^O_2_ quantum yield (Φ_Δ_ = 0.95). Consequently, in contrast to the oxygen radicals generated by the type I mechanism, ^1^O_2_ cannot be quenched by oxidative stress enzymes since ^1^O_2_ is not an oxygen radical but an energized molecular oxygen (E = 0.98 eV) [79,80].

**Figure 21:**
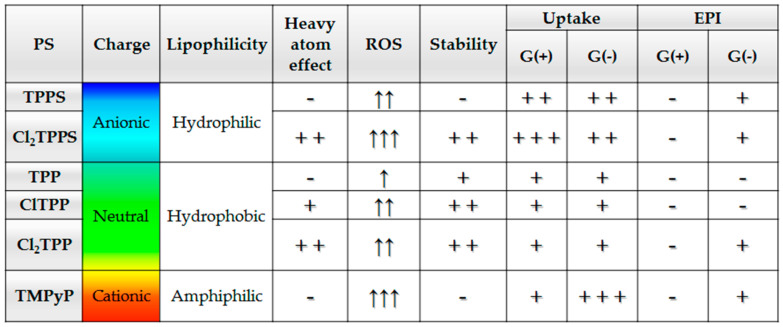
A summary of the factors influencing the efficacy of photodynamic photoinactivation. The structural modification of porphyrins and their influence on photophysical and biological activity in the performed experiments. Abbrev. In order: -/+/++/+++ or ↑/↑↑/↑↑↑ = no effect or the worst/moderate/most potent.

ROS interact with cells in various ways, and their response depends on many factors, such as (1) PS localization (attached to the bacteria cell wall or into cytosol); (2) type of generated ROS (type I and II); and (3) the mechanisms responsible for multidrug resistance (efflux pump inhibition, through EPIs). Several studies have demonstrated that an efflux pump inhibitor combined with the treatment is expected to (i) increase the intracellular concentration of the drugs that are expelled by the efflux pumps; (ii) decrease the intrinsic bacterial resistance to antibiotics (drugs); (iii) reverse the acquired resistance associated with efflux pump overexpression; and (iv) reduce the frequency of the emergence of resistant mutant strains [81]. Thus, combining PDI with an efflux pump inhibitor might improve the antibacterial effect of the therapy. Therefore, we have combined PDI mediated by porphyrin derivatives with verapamil—a recognized efflux pump inhibitor in bacteria, used as a reference in studies of drug efflux. The association of PDI with Ver decreases bacteria survival (especially for *E. coli*) in comparison with PDI alone. Combining the efflux pump inhibitor (Ver) with the PDI increases the reduction of bacteria viability—for *E. coli*, 3 logs of extra killing. In case of *S. aureus*, a reduction by 6 logs (>99.9999%) was achieved after each PDI scheme. Nevertheless, neither compound showed significant activity against Gram-negative *E. coli* in low-light doses (up to 20 J/cm^2^). Therefore, we examined their potentiation by the addition of potassium iodide. The data shows that PDI with TPPS, Cl_2_TPPS, and TMPyP was highly potentiated by KI (3–4 extra logs of killing), implying a contribution from hypoiodite and free iodine (Figure 8) [31]. Due to the high stability of the molecules modified with halogen atoms, their activity was also relatively higher at higher light doses. The results of these tests indicate that it is possible to obtain satisfactory results after doses lower than 60 J/cm^2^. Moreover, in the case of bacteria with a high pump activity, i.e., *E. coli*, these effects can be potentiated and achieved at lower doses if an EPI is used. Thus, these data suggest that in case of high-dose PDI, verapamil addition effectively improves bacteria inactivation. However, in case of low-dose PDI or limited photostability of the PS, the presence of KI and reactive iodide species gives more satisfactory results (Figure 21).

The strain-dependent differences in bactericidal photodynamic inactivation could be correlated to either the photosensitizer uptake levels or the efflux pump’s pharmacological inhibition. However, other factors may also affect the photodynamic efficacy, such as aggregation of the photosensitizer, the concentration of antioxidant enzymes, or the cellular repair proteins. However, all of these factors are not responsible for diminishing the PDI-induced antibacterial effect because, up to now, there is a lack of selection of photo-resistant bacteria after multiple photodynamic treatments.

## 4. Materials and Methods

### 4.1. Physicochemical Properties

#### 4.1.1. Chemicals

Commercially available *meso*-tetraphenyl porphyrin (TPP), *meso*-tetra(4-sulfonatophenyl)porphyrin as the tetrasodium salt (TPPS), 5,10,15,20-tetrakis(1-methyl-4-pyridinium)porphyrin tetra(p-toluenesulfonate) (TMPyP), and verapamil were obtained from Sigma-Aldrich. *Meso*-tetra(2-chlorophenyl)porphyrin (ClTPP), *meso*-tetra(2,6-dichlorophenyl)porphyrin (Cl_2_TPP) and *meso*-tetra(2,6-dichloro-3-sulfonatophenyl)porphyrin (Cl_2_TPPS) were prepared according to reported procedures [82]. All the other solvents, namely DMSO, DMF, and ethanol, were obtained from Sigma-Aldrich and used without further purification.

#### 4.1.2. UV-Vis-NIR Electronic Absorption Spectra Measurements

The photosensitizer samples were dissolved in DMSO. UV-Vis absorption spectra were recorded in quartz cuvettes (l = 1 cm) with a UV-3600 Shimadzu UV-Vis-NIR spectrophotometer in the range of 350–700 nm.

#### 4.1.3. Photostability Tests

The solutions of photosensitizers were prepared by diluting the porphyrin stock solution in DMSO in PBS (the DMSO content did not exceed 1%). The solutions of porphyrins with an absorbance ca. A = 1 were irradiated with 420 ± 20 nm LED (Instytut Fotonowy, Kraków, Poland) and with a halogen lamp with a 380 nm cut-off filter in a 1–120 J/cm^2^ light dose range. UV-Vis absorption spectra were recorded, and a decrease in the Soret band was investigated as a photodegradation process.

#### 4.1.4. Emission Spectra and Fluorescence Quantum Yield

Steady-state fluorescence emission spectra were recorded from 500 to 750 nm, with excitation at the Soret band (λ ≈ 420 nm) of each compound. The excitation and emission slits were both set to an 8 nm bandpass. Measurements were recorded with a FluoroLog-3 Spectrophotometer (Horiba Jobin Yvon). The samples were recorded in the following way: firstly, they were prepared with absorbance at the Soret maximum wavelength of around 0.2 and then diluted 100 times to measure fluorescence. Then, the sample was transferred into a fluorescence quartz cuvette to record the emission spectrum.

#### 4.1.5. Fluorescence Lifetime Measurements

The time-resolved fluorescence measurements were carried out using the Time-Correlated Single Photon Counting (TCSPC) mode from a FluoroLog-3 Spectrophotometer (Horiba Jobin Yvon). The samples were excited with 460 nm light from a ps pulsed LED as the MCS mode excitation source. The Instrument Response Function (IRF) was determined from a non-fluorescent suspension of colloidal silica (LUDOX 30%, Sigma Aldrich; St. Louis, MO, United States) in water, held in a quartz cuvette. Samples were prepared in DMSO with an absorbance around 0.1 at 420 nm and measured at room temperature. The decays were analyzed using Horia Jobin Yvon DAS-6 v6.4 analysis software and fitted with a double exponential model.

#### 4.1.6. LogP Determination

The partition coefficient was determined by the shake-flask method. Because the solvents partially dissolve in one another, a small amount of compound was dissolved in 5 mL PBS-saturated n-octanol. The sample was sonicated until all the compound dissolved. In the next step, 5 mL of PBS saturated with n-octanol was added and the experiment proceeded as before. The resulting mixture was vortexed for 15 min. The sample was centrifuged for 2 min at 3700 rpm to obtain an accurate phase separation. Next, 0.02 mL of each phase was taken and diluted in 3.98 mL DMSO. The next steps were a 5 min sonication and measurement of fluorescence spectra of the obtained solutions. The calibration curve was prepared in order to determine the concentration of the tested compounds in individual solutions. It was made using the fluorescence intensity of the compounds in series dilution solutions in DMSO with a 0.5% PBS buffer or n-octanol in the concentration range c = 1–100 nM.

### 4.2. Mechanistic Studies

#### 4.2.1. Detection of Singlet Oxygen with DMA

DMA (9,10-dimethyloanthracene) is a selective probe that reacts with singlet oxygen and converts into endoperoxide. This reaction enables the detection and estimation of $\Phi_{\Delta }$. DMA with porphyrins was dissolved in DMF and irradiated using an LED-light source 420 nm (±20 nm). A decrease in the DMA absorption spectra at 377 nm over irradiation with porphyrin confirmed the singlet oxygen generation. On the contrary, a blank sample (DMA in DMF) was also monitored and indicated no DMA photobleaching in the PS’s absence.

#### 4.2.2. Detection of Reactive Oxygen Species (ROS) with Fluorescent Probes

Hydroxyphenyl fluorescein (HPF) is a selective probe for hydroxyl radicals. Singlet Oxygen Sensor Green^®^ (SOSG) is a specific probe for singlet oxygen, and dihydroethidium (DHE) is a probe for the identification of the superoxide ion. These probes were employed for the detection of ROS after illumination of the PS. PS solutions were diluted to a final concentration of 10 μM per well. Next, each fluorescent probe was added to a well at a final concentration of 15 μM. PS solutions were irradiated with a 400 ± 20 nm LED light for various light doses. A microplate reader (Tecan Infinite M200 Reader) was used to measure the fluorescence intensity signal immediately before and after illumination with the appropriate excitation and emission parameters.

#### 4.2.3. Iodine Starch Test

Porphyrin solutions with absorbance ca. 1 au and 100 mM KI in PBS were irradiated with increasing light doses of 420 nm LED light. Aliquots (100 μL) were withdrawn after each light dose and addeded to the starch indicator solution (1% concentration, 100 μL). A microplate reader (absorbance at 600 nm) was used to measure the absorbance of the iodine complex. Controls were (1) PS + light + starch; (2) KI + starch + light; and (3) PS + KI + light but not presented in the results.

#### 4.2.4. Theoretical Calculations

The computations were carried out by using the Gaussian 9 code at the DFT and TDDFT levels of the theory. The ground state and structures optimization were done using the M06 exchange and correlation functional coupled with the 6-31G(d) basis set. The absorption spectra, singlet-triplet energy gaps, and HOMO–LUMO levels were computed using the B3LYP exchange-correlation functionals with the polarizable continuum model (PCM), which considers solvent effects (water ε = 78.54). M06/6-31G(d) and B3LYP/6-31G(d) were chosen according to previous studies [83,84]. The visualization of molecular structures and orbital contour plots were carried out using the Molden visualization software. Simulation of absorption spectra was carried out from 20 low-lying excited states calculated with a Gaussian distribution. The UV-Vis spectra were generated with the Molden software by considering the full-width at half-maximum as 10 nm. The molecular electrostatic potential was calculated with Gaussian software and visualized with Gabedit software.

#### 4.2.5. Cyclic Voltammetry and Spectroelectrochemistry Measurements

Cyclic voltammetry was carried out in a three-electrode system with a carbon, platinum wire, and Ag/Ag^+^ electrode (AgNO_3_ (10 mmol/dm^3^) in 0.1 mol/dm^3^ Bu_4_NClO_4_ in acetonitrile) as the working, counter, and the reference electrodes, respectively. The silver pseudo-reference electrode was calibrated by a reference redox system (ferrocene). The studied porphyrins were dissolved in 0.1 mol/dm^3^ Bu_4_NClO_4_ in DMSO. The electrode potential was controlled by an electrochemical analyzer Autolab PGSTAT302N. The cyclic voltammetry was performed between −2.5 V and 1.0 V vs. Ag/Ag^+^ at a scan rate of 50 mV/s. The spectroelectrochemistry, based on electrochemical measurements combined with UV−Vis spectroscopy, was conducted for the same solution of porphyrins. The electrochemical measurements were carried out in a three-electrode cell, with platinum wire and Ag/Ag^+^ as a counter and the reference electrode, respectively. As the working electrode was used platinum mesh. The electrode potential was controlled by an electrochemical analyzer Autolab PGSTAT302N. The applied potential was lowered every 90 s by 50 mV. The absorption was recorded by a UV–VIS 8453 Diode Array Hewlett-Packard.

### 4.3. Biological Studies

#### 4.3.1. Bacterial Strains and Culture Conditions

The Gram-positive bacterium *S. aureus* and Gram-negative bacterium *E. coli* were used in the study. *S. aureus* (8325-4) and *E. coli* (K12) cells were cultured in brain heart infusion (BHI) broth and LB broth, respectively, with aeration at 37 °C under shaking conditions in an orbital incubator (180 rpm). Cell growth was assessed with absorbance measurements at 600 nm (OD600) until the absorbance reached 0.5, which corresponded to approximately 10^7^ CFU per mL.

#### 4.3.2. Binding of the Photosensitizer to Microorganisms—Uptake Studies

The microorganisms were incubated in suspension with a photosensitizer (20 µM) for selected time intervals (0–120 min) in the dark at RT. The unbound photosensitizer was removed by washing twice in PBS without Ca^2+^ and Mg^2+^. After the second wash, bacteria were lysed in 10% SDS for 24 h. The cellular uptake of the photosensitizer was evaluated by determination of fluorescence using excitation at the Soret band (420 nm) and emission between 630–700 nm (Tecan Infinite M200 Reader). Calibration curves were prepared in 10% SDS and used for the determination of PS concentration. Uptake values were obtained by dividing the PS concentration by the number of CFU. The number of molecules per cell was calculated from the PS concentration and calibration curve.

Cellular uptake of the investigated photosensitizers was also determined using flow cytometry and quantified based on the porphyrin’s red fluorescence. For this analysis, *S. aureus* and *E. coli* (0.5 × 10^6^ cells) were incubated with each porphyrin at 20 µM and APF 25 µM for 2 h. After this incubation, cells were washed two times with HBSS and harvested for analysis. Then, the cells were collected by centrifugation and then resuspended in 200 µL of PBS. Stained cells were then examined using Guava^®^ easyCyte™ flow cytometer equipped with a 488 nm laser. The obtained data were analyzed using InCyte software (MerckMillipore, Burlington, MA, USA).

#### 4.3.3. ROS Generation In Vitro

ROS generation in vitro was investigated using dual-color flow cytometry analysis quantified after 2 h incubation of bacteria with each photosensitizer (20 µM). After this incubation, cells were washed with PBS (without Ca^2+^ and Mg^2+^) and then incubated with APF (25 µM) prepared in PBS for the next 2 h. After this time, cells were washed twice with PBS and irradiated with 10 J/cm^2^ blue light (LED diode, 420 ± 20 nm, Instytut Fotonowy, Kraków, Poland). studies [85]. After irradiation, cells were collected by centrifugation and resuspended in 1 mL of PBS examined using BD Accuri™ C6 flow cytometer. The obtained data were analyzed using BD Software (BD Bioscience, Bergen, NJ, USA).

#### 4.3.4. Laser Scanning Confocal Microscopy (LSCM) Imaging

Accumulation of the selected porphyrin derivatives in the microorganisms was followed with confocal imaging using a Zeiss LSM880 laser-scanning microscope equipped with an argon-ion laser. The objective was a water immersion “dipping” lens (100×, Carl Zeiss Ltd., Jena, Germany) with a working distance of 1.46 mm. Accordingly, for the uptake studies, bacteria were incubated with the photosensitizer solution (20 μM) for an appropriate time interval determined in the uptake studies (2 h). After washing, the bacteria samples were counterstained with Hoechst33342 (10 µg/mL) for 10 min, placed on the microscopic glass slides, and imaged. Registered images were analyzed with the Zeiss ZEN software.

#### 4.3.5. Efflux Pump Activity—MDR Resistance Assay

The activities of MDR1 in cells were characterized by the Vybrant Multidrug Resistance Assay Kit V-13180 (Molecular Probes, Eugene, OR, USA). In this test, the effects of modulators on P-gp activity were determined by measuring the calcein-acetoxymethyl ester (calcein AM) fluorescence in bacteria treated with a photosensitizer and photosensitizer with verapamil. Briefly, cells were treated with each photosensitizer and incubated for 2 h. A total of 100 µM verapamil was added to the selected samples, mixed, and incubated at 37 °C for 15 min. After this incubation, calcein AM was added with a final concentration of 0.25 µM. The samples were mixed and incubated at 37 °C for the next 15 min. After this time, samples were centrifuged for 5 min at 200× *g*, the supernatant was removed, and the bacteria were resuspended in 200 µL of cold (4 °C) PBS. The washing step was repeated two more times. The calcein retention measurement was performed using a Guava^®^ easyCyte™ flow cytometer equipped with a 488 nm laser. The obtained data were analyzed using InCyte software (MerckMillipore, Burlington, MA, USA).

#### 4.3.6. Photoinactivation of the Microorganisms (PDI)

The tested microorganisms were incubated with a 20 µM solution of each photosensitizer for 2 h in the dark at room temperature. In the case of verapamil or KI-treated groups, KI (100 mM) or verapamil hydrochloride (100 µM) were added to the PS solution. Then, aliquots (1 mL) were transferred to a 12-well plate and illuminated with a blue light at various doses 0–100 J/cm^2^ using an LED (Instytut Fotonowy, Poland). After illumination (or dark incubation—control), samples were mixed, serial-diluted in PBS, and plated (LB agar) to determine the number of CFUs. Cells treated only with light (no photosensitizer) were as viable as untreated cells (data not shown). The viability was also monitored using the LIVE/DEAD *Bac*Light Bacterial Viability Kit (Invitrogen; monitors membrane integrity) according to the manufacturer’s instructions. In order to visualize the PDI effect, images of *E. coli* and *S. aureus* colonies grown on LB (*E. coli*) or BHI (*S. aureus*) agar plates were registered using Azure 600 Imaging System (Azure Biosystems, Dublin, CA, USA).

#### 4.3.7. Scanning Electron Microscopy (SEM) Imaging

The bacteria images were taken by scanning electron microscopy (SEM), using a Tescan VEGA 3 with a LaB_6_ emitter. The measurements were performed on a carbon sheet without gold sputtering. Observations were performed in high-vacuum conditions with low voltage (2 kV).

#### 4.3.8. Statistical Analysis

The obtained data are presented as the mean and standard deviation (of the mean). Using Student’s *t*-test, we performed the two-group comparisons. Statistical analysis was performed with STATISTICA 13.

## 5. Conclusions

This paper discusses the physicochemical properties and examines the antibacterial activity of six different porphyrins, namely, TPP, ClTPP, Cl_2_TPP, TPPS, Cl_2_TPPS, and TMPyP, which have not been studied in such a comprehensive approach so far. The introduction of the electron-withdrawing chlorine atoms improves the optical properties, reduces aggregations, and enhances photostability. The presence of negatively or positively charged groups in the peripheral position increases the solubility and amphiphilicity of the compounds and consequently increases electrostatic interaction with the biological membrane. The molecular charge distribution and lipophilicity have a significant impact on the accumulation of porphyrin-based photosensitizers. However, the biological activity of photosensitizers is not easy to predict based just on their physico-chemical characteristics. For example, TMPyP, which exhibits excellent photophysical properties, may not be effective in bacteria-killing during a long time of irradiation due to its higher lability. In turn, Cl_2_TPPS, whose appropriate redox properties ensure its stability even when using high light doses, turns out to be the most effective PS for photoinactivation of bacteria. This leads to the conclusion that predicting PDI activity on photochemical studies (e.g., quantum yields of ISC and singlet oxygen) does not allow to select the most active molecules. Potentiation of the PDI protocol by KI addition with short- and long-lived iodine species generation may enhance photokilling, especially in Gram-negative bacteria with increased resistance. Moreover, PDI and other alternative antimicrobial strategies may provide a niche for EPI implementation. Optimizing PDI with efflux pump inhibition, its efficacy may be enhanced and sets a new trend for combined treatments against MDR bacteria. Our work demonstrates that only a complete analysis of optical, photochemical, redox and pharmacological properties allows to make a proper selection of the most appropriate photosensitizer for effective PDI. Additionally, for photosensitizers with lower antimicrobial activity, it is possible to modulate the protocol (with or without KI and verapamil) for the effective photoinactivation of bacteria.

## Figures and Tables

**Table 1:** Optical and photochemical properties of the porphyrins determined in DMSO.

Absorption						Emission			
λ (nm), ε (M^−1^ cm^−1^) 10^3^						λ (nm), Φ_F_, τ_F,_ Φ_Δ_			
Compound	B	Q_y_ (0–1)	Q_y_ (0–0)	Q_x_ (0–1)	Q_x_ (0–0)	(0–0) (0–1)	Φ_F_	τ_F_/ns	Singlet Oxygen Quantum Yield
TPP	418 195.3	513 7.9	549 3.4	590 2.2	645 -	648, 714	0.10	10.1	0.60
ClTPP	417 234.7	512 15.3	542 4.5	587 3.1	652 -	651, 712	0.04	7.2	0.65
Cl_2_TPP	418 258.6	511 15.3	541 -	588 -	657 -	649, 714	0.02	4.9	0.83
TPPS	420 205.3	515 8.6	550 6.9	590 -	646 -	650, 715	0.10	11.5	0.70
Cl_2_TPPS	419 240.5	513 17.0	544 5.3	587 3.4	642 2.7	648, 711	0.02	6.0	0.95
TMPyP	425 190.3	521 6.9		585 1.9	640	664, 714	0.05 *	4.6 **	0.70

* [58], ** [59].

**Table 2:** Energy gaps (∆E_Sn−Tm_ eV) between the involved excited states calculated at the B3LYP/cc-pVDZ/M06/6−31G(d) levels of theory.

	TPP	ClTPP	Cl_2_TPP	TPPS	Cl_2_TPPS	TMPyP
∆E_S1-T1_	0.75	0.78	0.78	0.75	0.79	0.70
∆E_S1-T2_	0.52	0.49	0.47	0.51	0.48	0.43
∆E_S3-T1_	1.70	1.71	1.69	1.68	1.70	1.51
∆E_S3-T2_	1.47	1.42	1.39	1.44	1.40	1.24

**Table 3:** LogP values determined for all the tested porphyrins by the shake-flask method.

Compound	LogP
TPP	3.02
ClTPP	3.09
Cl_2_TPP	3.12
TPPS	−1.20
Cl_2_TPPS	−1.08
TMPyP	2.06

## Supplementary Materials

Supplementary Materials can be found at https://www.mdpi.com/1422-0067/21/22/8716/s1.

## Abbreviations

ABC	ATP-binding cassette
CFU	Colony forming unit
DFT	Density functional theory
DMMB	1,9-dimethylmethylene blue
DNA	Deoxyribonucleic acid
EPI	Efflux pump inhibitor
HOMO	Highest occupied molecular orbital
ISC	Intersystem crossing process
LUMO	Lowest unoccupied molecular orbital
LPS	Lipopolysaccharides
MB	Methylene blue
MDR	Multidrug resistance
MEP	Microbial efflux pump
NHE	Normal hydrogen electrode
NSC	Normal standard electrode
PDI	Photodynamic inactivation
PDT	Photodynamic therapy
PS	Photosensitizer
RB	Rose Bengal
ROS	Reactive oxygen species
SAR	Structure–activity relationship
SCE	Saturated calomel electrode
TBO	Toluidine blue O
TDDFT	Time-dependent density functional theory
