# Supplementary material for: Photodynamic Inactivation of Bacteria with Porphyrin Derivatives: Effect of Charge, Lipophilicity, ROS Generation, and Cellular Uptake on Their Biological Activity In Vitro
Source: Int J Mol Sci. 2020 Nov 18;21(22):8716. doi: 10.3390/ijms21228716 (PMC7698881; doi:10.3390/ijms21228716)
Supplement: Supplementary file 1 [file ijms-21-08716-s001.pdf]

International Journal of Molecular Sciences

Supporting Information for

Photodynamic inactivation of bacteria with porphyrin derivatives: effect of charge, lipophilicity, ROS generation and cellular uptake on their biological activity *in vitro*

Adam Sułek [1]†, Barbara Pucelik [2]†, Marcin Kobielusz [1], Agata Barzowska [2] and Janusz M. Dąbrowski [1,]*

[1] Faculty of Chemistry, Jagiellonian University, 30-387 Krakow, Poland
[2] Małopolska Center of Biotechnology, Jagiellonian University, 30-387 Krakow, Poland
† These authors contributed equally to this work.
* Correspondence: jdabrows@chemia.uj.edu.pl; Tel.: +48-12-686-2488; Fax: +48-12-686-2750

1. Spectroscopic characterization of photosensitizers

Molar absorption coefficients were determined for each compound using PS solutions in DMSO in concentrations ranging from 10^{-7} to 10^{-6} M.

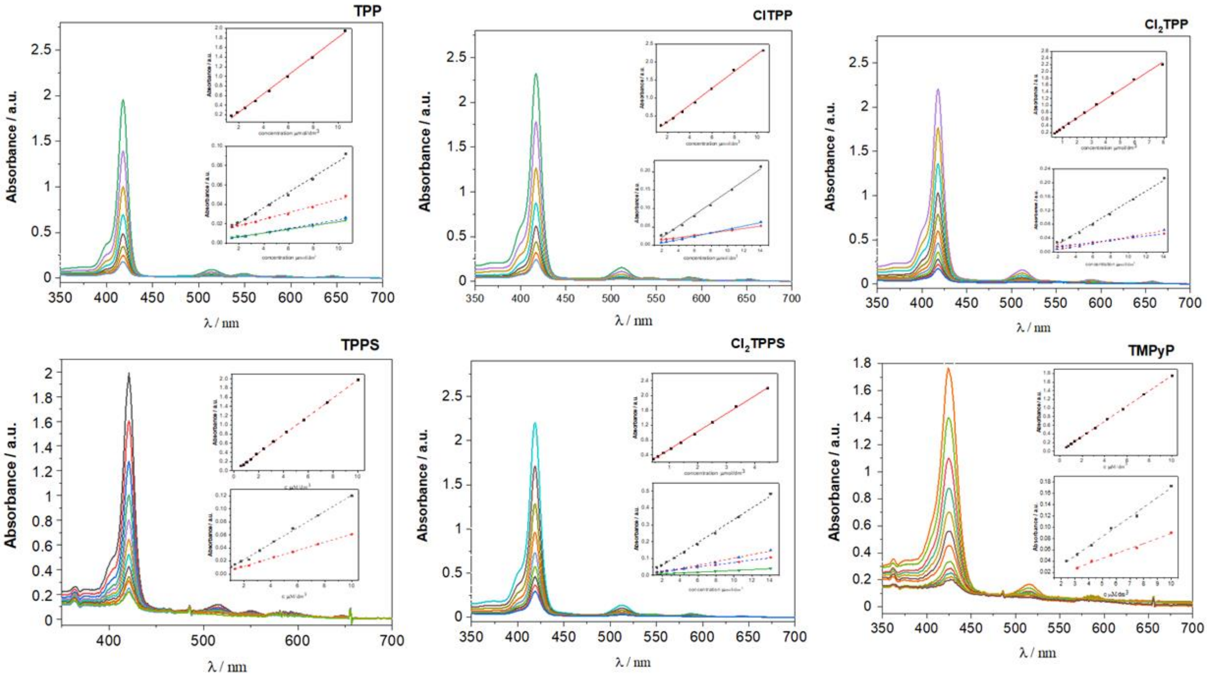

Figure S1. The absorption spectra for series of studied porphyrins with different concentration. The linear curves are based on the absorption value used to determine the molar absorption coefficient. The results are given in Table 1.

2. Fluorescence quantum yields and lifetime

Fluorescence quantum yields (Φ_F) were calculated by comparison of the integrated are below each emission spectrum with that of TPP (Φ_F=0.10 in toluene), according to equation S1.

$$\phi_{F\,sample} = \phi_{F\,ref} \times \frac{F_{sample}}{1-10^{-Abs_{sample}}} \times \frac{1-10^{-Abs_{ref}}}{F_{ref}} \times \frac{\eta^2{}_{sample}}{\eta^2{}_{ref}} \quad (S1)$$

where:

F_{sample} stands for the integration area under the emission spectrum,

Abs is the absorbance at the excitation wavelength,

η is the refractive index of the solvent used

The determined fluorescence lifetime (τ_F) of the first excited singlet state for each porphyrin is found to be better fitted as double exponential. All fits presented χ^2 values smaller than 1.3, indicating good fitness of the model to the data.

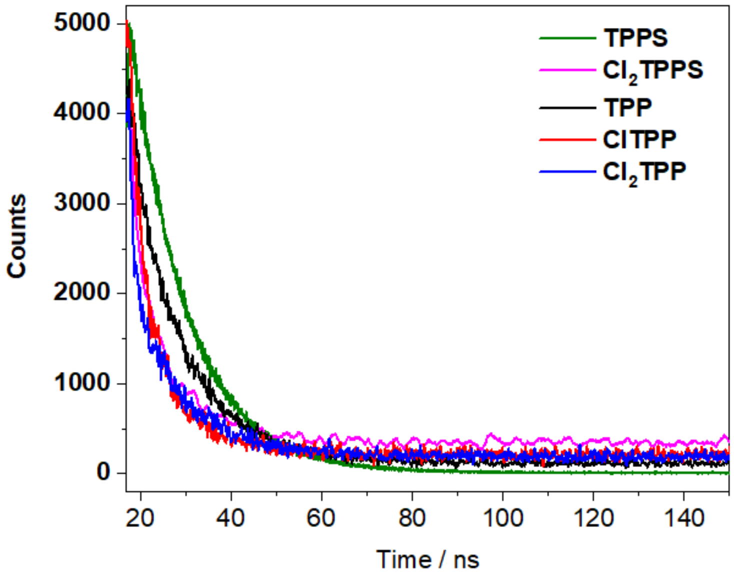

Figure S2. Fluorescence decay curves in ethanol at room temperature of porphyrins (abs 0.1 at 420 nm) at emission wavelength 650 nm. All the measurements were carried out at an excitation wavelength of 420 nm.

3. Theoretical calculation

As a first step of the work was optimized the structures of the *meso*-tetraphenyl porphyrins, their chlorinated derivatives and sulfo-derivatives. The calculations are in accordance with other reported for TPP and TPP metallo-derivatives [1, 2]. The mixed electronic transitions between the two highest occupied orbitals (HOMO and HOMO-1) and the two lowest unoccupied orbitals (LUMO and LUMO+1) are mainly responsible for both the strong Soret bands (B) and weaker Q bands in the UV-Vis region. The singlet and triplet excited states with oscillator strength and molecular orbital contribution have been computed using the B3LYP exchange-correlation functionals (Table S1).

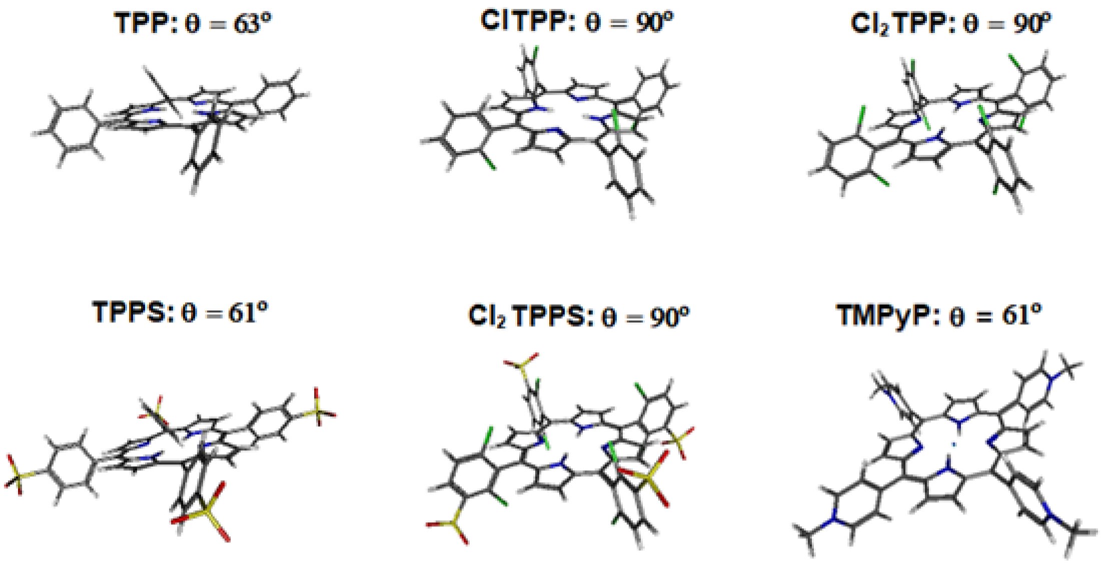

Figure S3. The graphical representation of calculated porphyrins ground geometry calculated at M06/6-31G(d) level of theory with torsion angel value   highlighted.

Table. S1. Main vertical excitation energies (ΔE), absorption wavelengths (λ), oscillator strengths (f), and transitions (Molecular Orbital contribution in %) for the studied compounds in PCM theory (water environment), computed at the M06/6−31 + G * level of theory.

**Compound**	**Excited state**	**λ_{theo} (nm)**	**ΔE (eV)**	**f**	**MO contribution (>10%)**
TPP	S_1	577	2.15	0.06	H→L 70%; H-1→L+1 30%
	S_2	541	2.29	0.08	H→L+1 69%; H-1→L 32%
	S_3	399	3.15	1.35	H-1→L+1 62%; H→L 26%
	S_4	392	3.28	1.68	H-1→L 65%; H→L+1 33%
	T_1	889	1.40	0.00	H→L+1 87%; H-1→L 13%
	T_2	760	1.63	0.00	H→L 98%
ClTPP	S_1	558	2.22	0.01	H→L+1 62%; H-1→L+1 37%
	S_2	523	2.37	0.01	H→L 58%; H-1→L 42%
	S_3	393	3.15	1.10	H-1→L 56%; H→L 29%; H-3→L 16%
	S_4	382	3.24	1.59	H-1→L+1 56%; H→L+1 42%
	T_1	860	1.44	0.00	H→L 83%; H-1→L 19%
	T_2	716	1.73	0.00	H→L+1 96%
Cl_2TPP	S_1	559	2.22	0.01	H→L+1 60%; H-1→L+1 38%
	S_2	524	2.36	0.01	H-5→L 56%; H-1→L 42%
	S_3	395	3.13	1.06	H-5→L+1 53%; H→L 30%; H-3→L 17%
	S_4	383	3.23	1.54	H-1→L 55%; H→L+1 42%
	T_1	862	1.44	0.00	H→L 80%; H-1→L 21%
	T_2	708	1.75	0.00	H-1→L 93%
TPPS	S_1	580	2.14	0.07	H→L 70%; H-1→L+1 29%
	S_2	544	2.27	0.11	H→L+1 70%; H-1→L 31%
	S_3	403	3.07	1.54	H-1→L 63%; H→L 26%
	S_4	397	3.12	1.80	H-1→L+1 65%; H→L+1 30%
	T_1	889	1.39	0.00	H→L 85%; H-1→L 13%
	T_2	760	1.63	0.00	H→L+1 95%
	S_1	561	2.21	0.01	H→L+1 60%; H-1→L+1 38 %

Cl_2TPPS	S_2	526	2.35	0.01	H→L 58%; H-1→L 41%
	S_3	397	3.12	1.14	H-1→L 53%; H→L 30%; H-11→L 16%
	S_4	386	3.21	1.61	H-1→L+1 56%; H→L+1 41%
	T_1	868	1.43	0.00	H→L 80 %; H-1→L 20 %
	T_2	714	1.73	0.00	H→L+1 93%
TMPyP	S_1	562	2.21	0.00	H-1→L+1 34%; H→L 57%
	S_2	529	2.34	0.00	H-1→L 50%; H→L+1 45%
	S_3	502	2.47	0.01	H→L+2 85%
	S_{11}	410	3.02	0.71	H-3→L 30%; H-1→L+1 33%; H→L 21%
	S_{13}	394	3.15	1.24	H-1→L 35%; H→L+1 37%
	T_1	823	1.51	0.00	H-1→L 36%; H→L+1 50%
	T_2	696	1.78	0.00	H-1→L+1 12%; H→L 78%

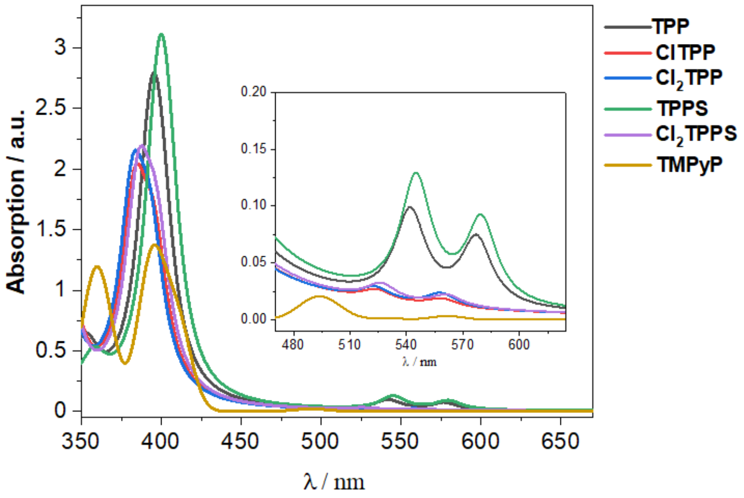

Figure S4. Normalized electronic absorption spectra of porphyrins calculated at B3LYP/cc-pVDZ/M06/6−31G(d) level of theory.

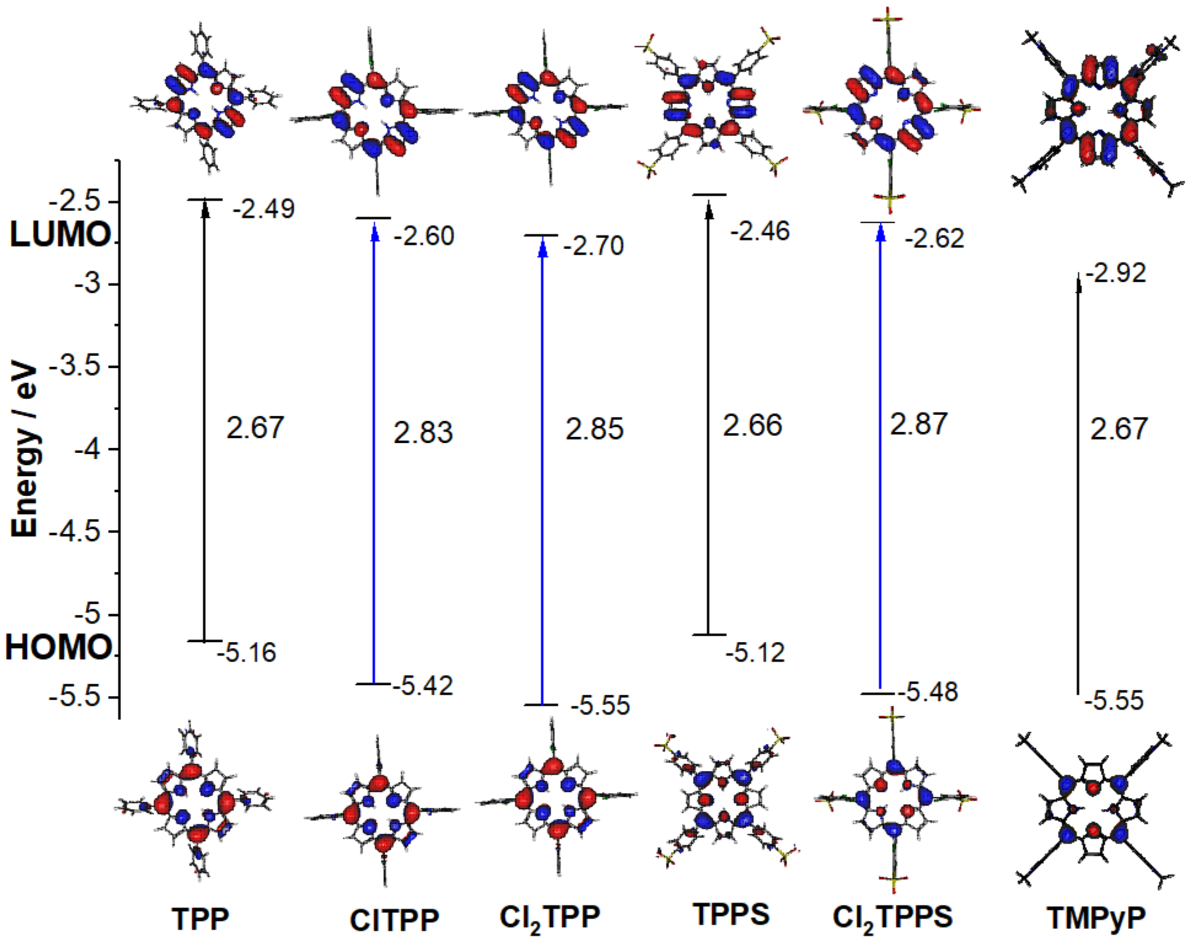

Figure S5. Orbital-correlation diagram of HOMO and LUMO for porphyrins with blue shifted highlighted.

4. Cyclic voltammetry

The measurement of Bu_4NClO_4 in DMSO (pure solvent with electrolyte 0.1 M) shows an electrochemical window in range –2.5 V up to 1.0 V without reduction or oxidation of solvent.

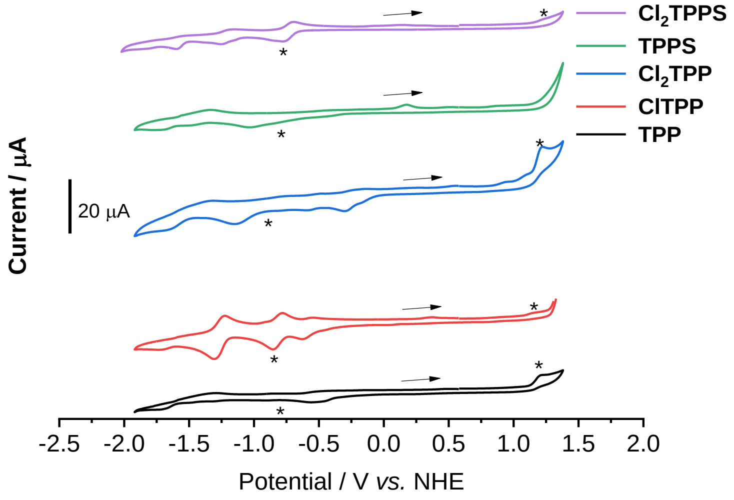

Figure S6. The cyclic voltammograms of porphyrins. The solvent is DMSO and the concentration is ca. 0.5 mM in 0.2 M TBAP 20 mV/s. vitreous carbon working electrode.

Table S2. Cyclic voltammograms halfwave value of tested porphyrins.

	Ered	Eox	$E_{1/2}$	Ered	Ered	Eox	$E_{1/2}$	Ered	Ered	Eox	$E_{1/2}$	Ered	Eox
TPP	–1.75			–1.49	–1.33	–1.31	–1.32		–0.89	–0.86	–0.88		
ClTPP	–1.72				–1.30	–1.22	–1.26		–0.84	–0.78	–0.81		
Cl_2TPP	-1.83			–1.60	–1.38			–1.24	–0.85			–0.77	
TPPS	–1.69					–1.35		–1.15	–0.80				–0.74
Cl_2TPPS	–1.74	–1.65	–1.70	–1.53		–1.33		–1.06	–0.68				

	Ered	Eox	$E_{1/2}$	Ered	Eox	Ered	Eox	$E_{1/2}$	Ered	Ered	Eox	$E_{1/2}$	Ered
TPP	–0.55				–0.13	0.25			0,49				
ClTPP	–0.61	–0.55	–0.58			0.08			0.38	0.84	0.90	0.87	1.14
Cl_2TPP	–0.49					0.08							
TPPS	–0.58	–0.48	–0.53	–0.29	–0.15	0.21	0.27	0.24	0.55	0.73	0.94	0.83	
Cl_2TPPS	–0.46				–0.16		0.17		0.50		0.86		1.01

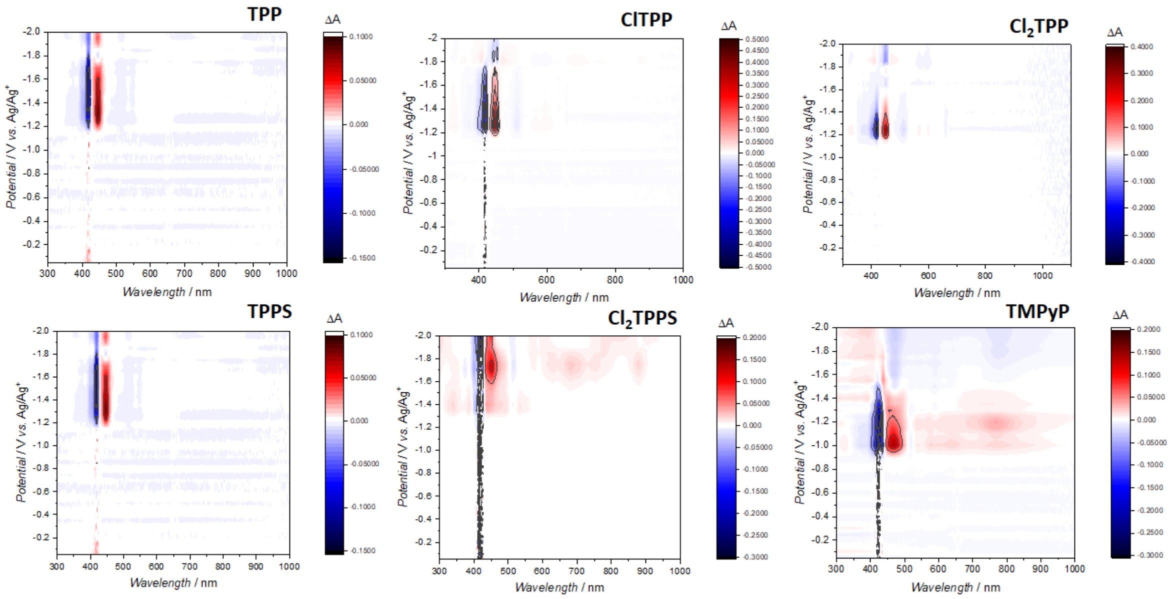

Figure S7. The absorbance change as a function of applied potential (vs Ag/Ag+) and wavelength.

Table S3. The excited state potential calculated with spectroelectrochemical method and cyclic voltametry.

compound	spektroelectrochemical measurment	CV measurment
TPP	–0.57	–0.55
ClTPP	–0.57	–0.61
Cl_2TPP	–0.51	–0.49
TPPS	–0.62	–0.58
Cl_2TPPS	–0.72	–0.68

5. Singlet oxygen quantum yields

$$\phi_{\Delta} = \phi_{\Delta Std} \frac{R I_{Std}}{R_{Std} I} \quad (S2)$$

where:

$\phi_{\Delta Std}$ is the quantum yields of singlet oxygen formation for the standard,

R is the DMA photobleaching rates in the presence of porphyrins and R_{Std} in the present of the standard (TPPS),

I and I_{Std} are the rates of light absorbed by the sample and the standard

6. Photostability

The stability of tested compound and their photobleaching rates should be measured under photoinactivation conditions (420 nm LED) but also under whole visible range of irradiation. For this reason we also check theire photobleaching with >380 nm of irradiation.

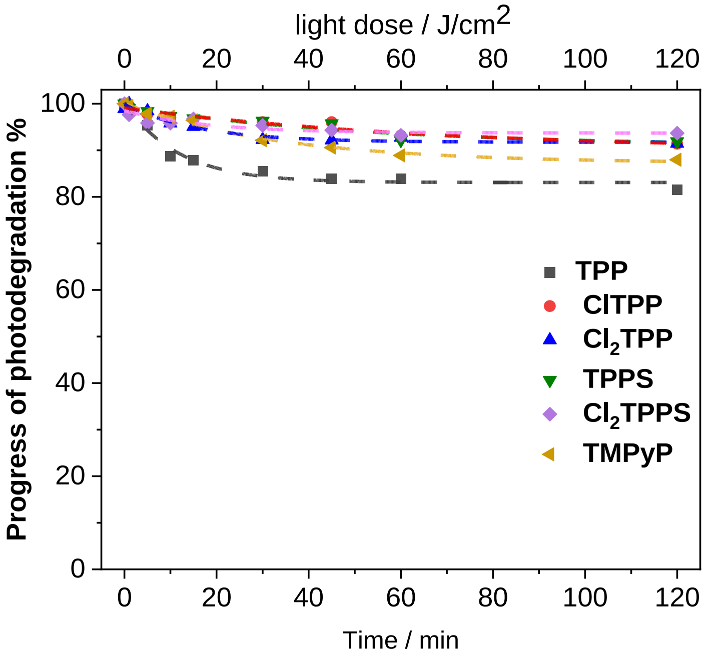

Figure S8. The photostability of porphyrins: TPP, ClTPP, Cl_2TPP, TPPS, Cl_2TPPS and TMPyP in PBS solution. Irradiation of the solution was carried out using 75 W xenon lamp through a water filter and 380 cut-off filters.

7. Photodynamic inactivation of microorganisms

For better visualization of the bactericidal effect, aliquots of control (Figure S9) or photodynamically-treated *E. coli* (Figure S10-S15) and *S. aureus* (Figure S16-S21) suspensions on agar plates are presented below. It clearly shows colony forming units in the absence of PDI or when exposed to the photosensitizer (dark toxicity), but not when exposed to high blue light dose with PS and PS+Ver.

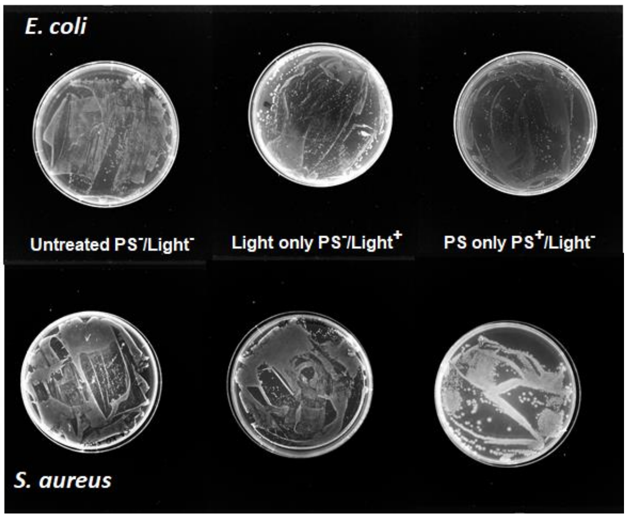

Figure S9. The control aliquots samples of *E. coli* (upper part) and *S. aureus* (lower part) untreated, treated with PS alone and treated with light (100 J/cm^2) alone.

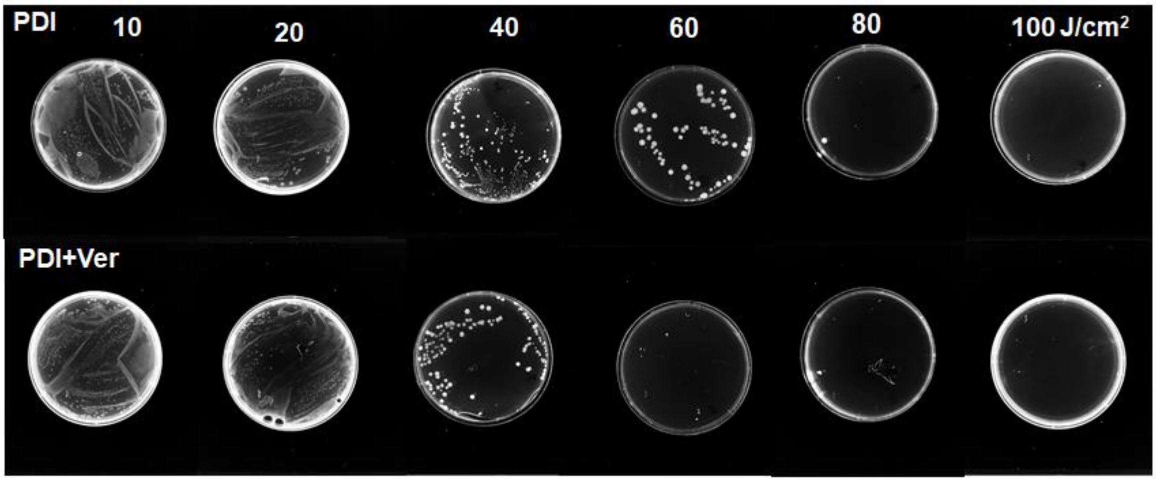

Figure S10. The aliquots samples of *E. coli* treated with TmPyP with various light dose 10 - 100 J/cm^2.

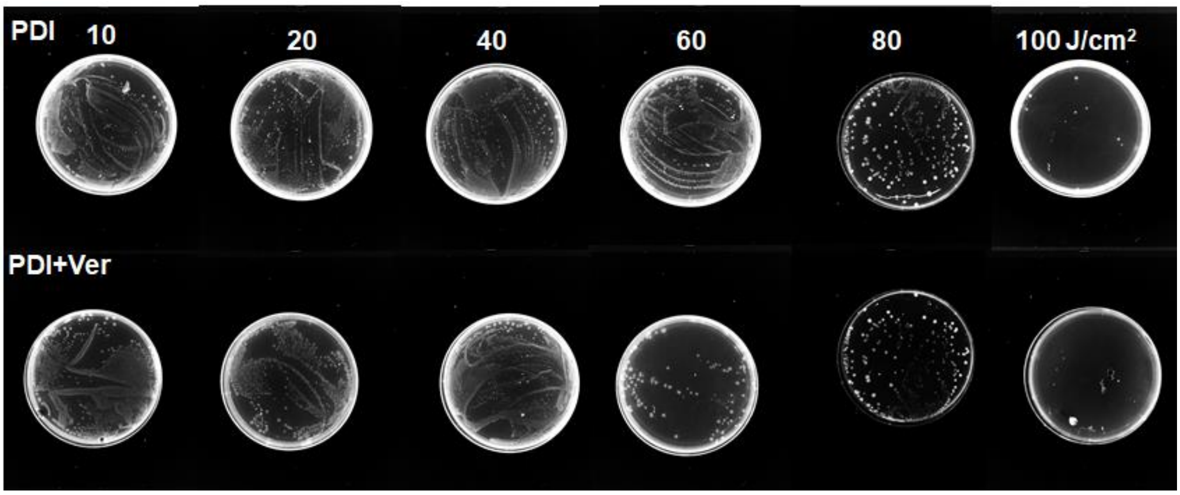

Figure S11. The aliquots samples of *E. coli* treated with TPPS with various light dose 10 - 100 J/cm^2.

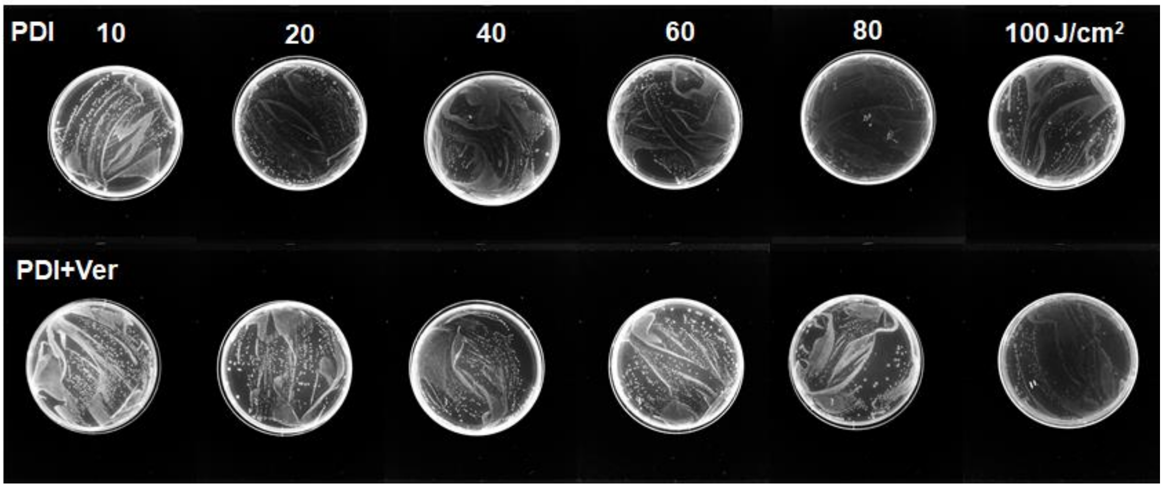

Figure S12. The aliquots samples of *E. coli* treated with TPP with various light dose 10 - 100 J/cm^2.

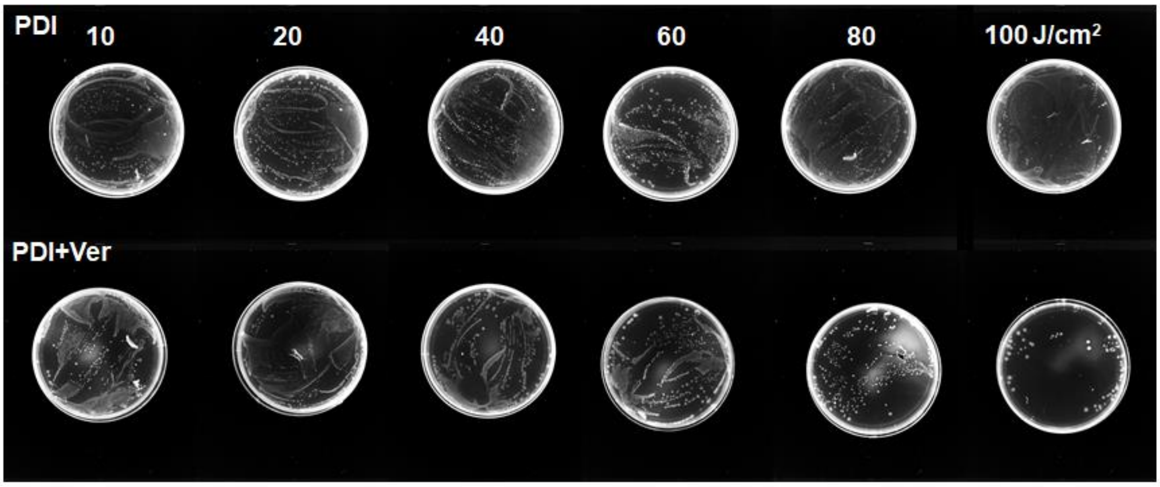

Figure S13. The aliquots samples of *E. coli* treated with ClTPP with various light dose 10 - 100 J/cm^2.

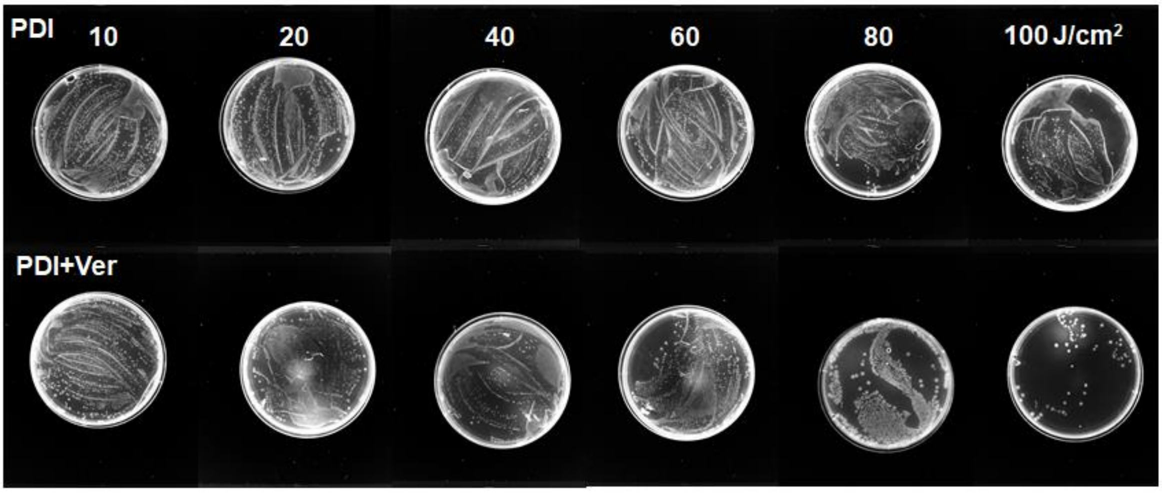

Figure S14. The aliquots samples of *E. coli* treated with Cl_2TPP with various light dose 10 - 100 J/cm^2.

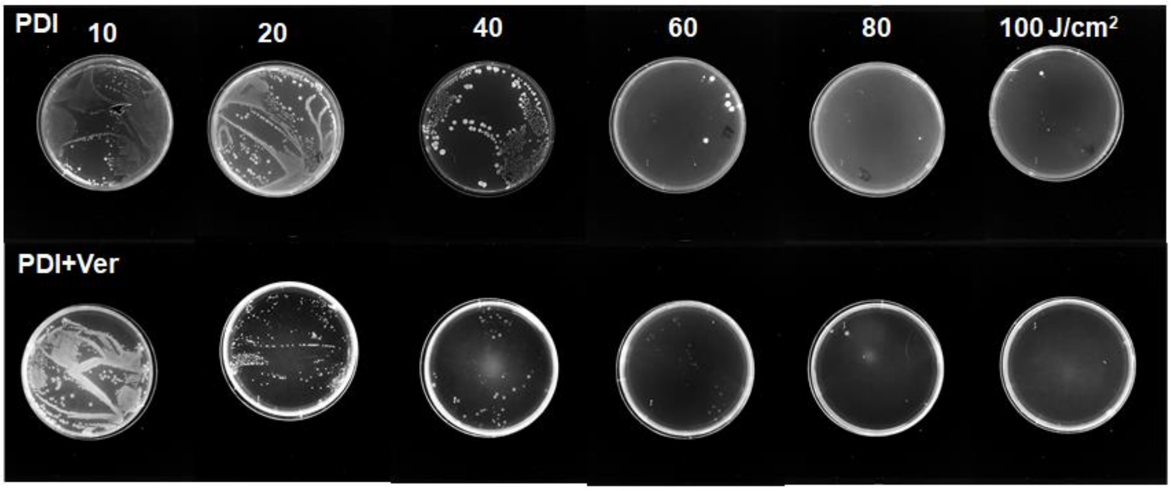

Figure S15. The aliquots samples of *E. coli* treated with Cl_2TPPS with various light dose 10 - 100 J/cm^2.

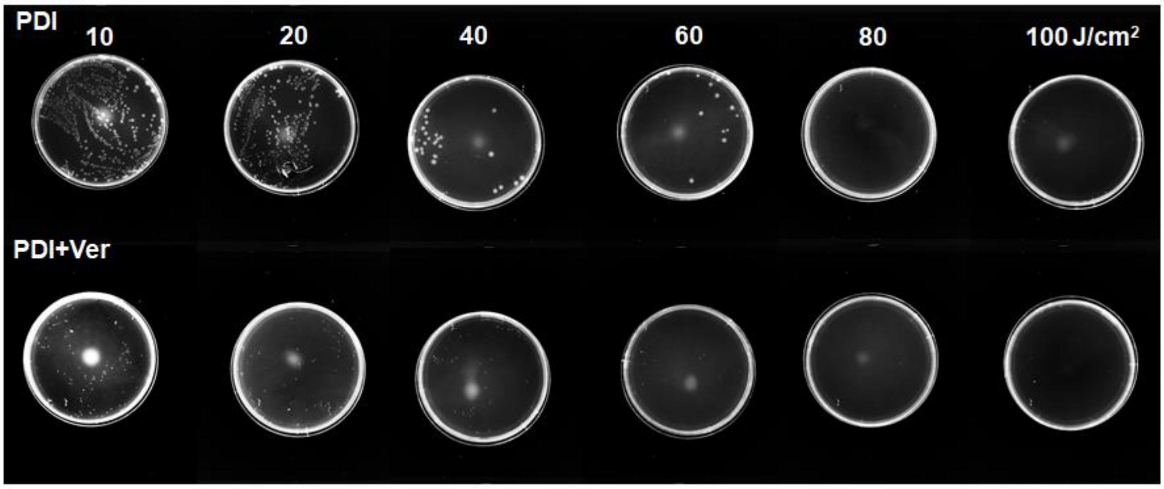

Figure S16. The aliquots samples of *S. aureus* treated with TMPyP with various light dose 10 - 100 J/cm^2.

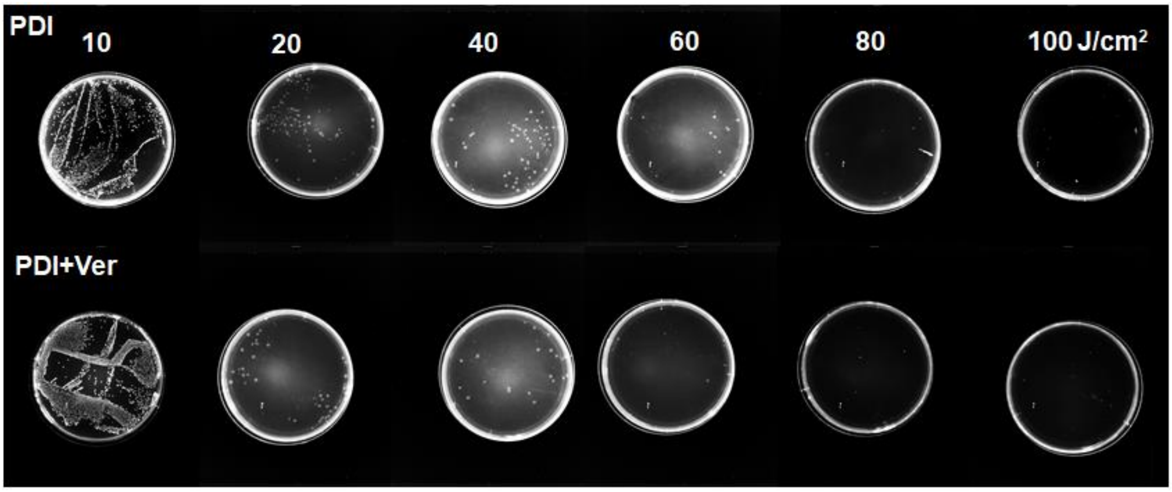

Figure S17. The aliquots samples of *S. aureus* treated with TPPS with various light dose 10 - 100 J/cm^2.

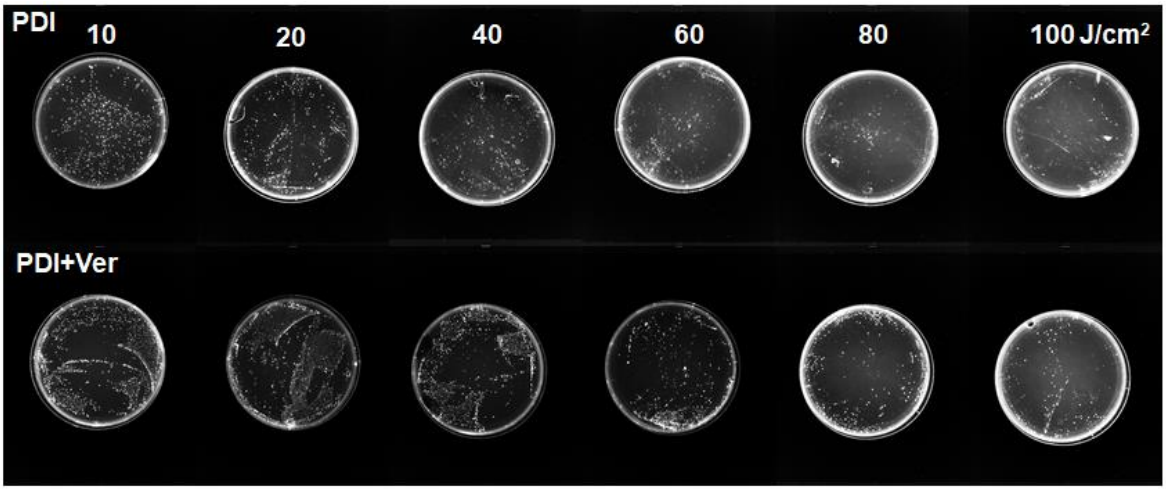

Figure S18. The aliquots samples of *S. aureus* treated with TPP with various light dose 10 - 100 J/cm^2.

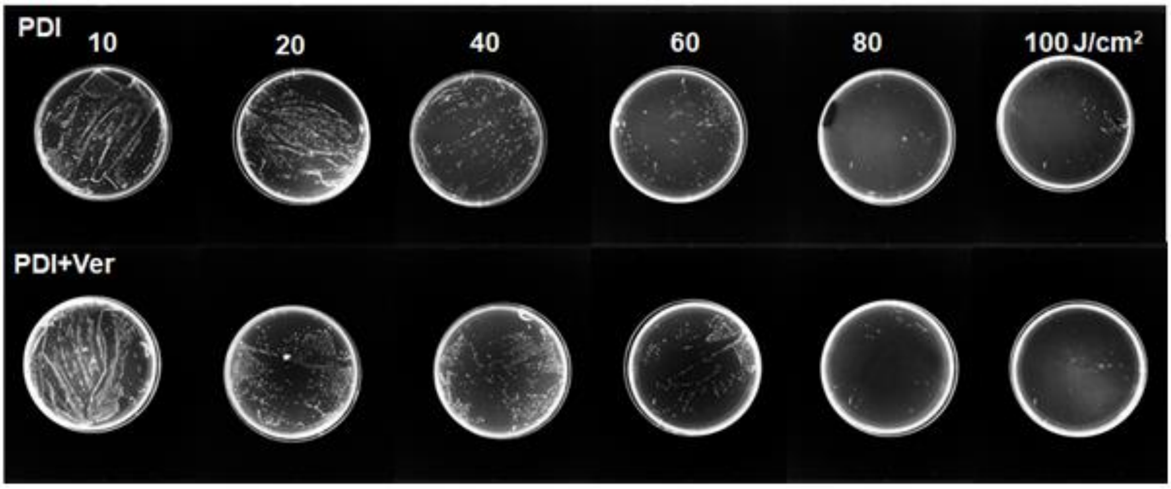

Figure S19. The aliquots samples of *S. aureus* treated with ClTPP with various light dose 10 - 100 J/cm^2.

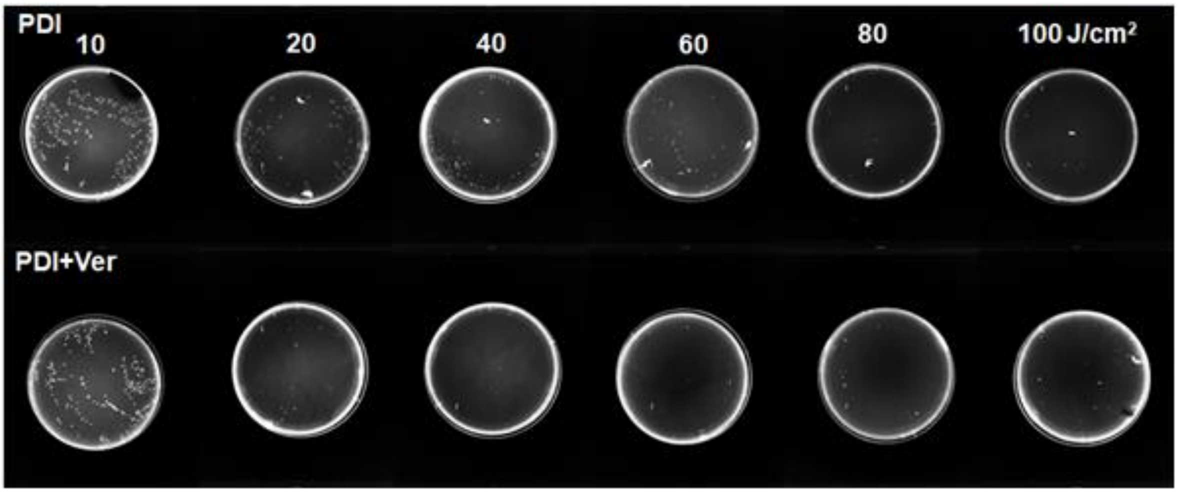

Figure S20. The aliquots samples of *S. aureus* treated with Cl_2TPP with various light dose 10 - 100 J/cm^2.

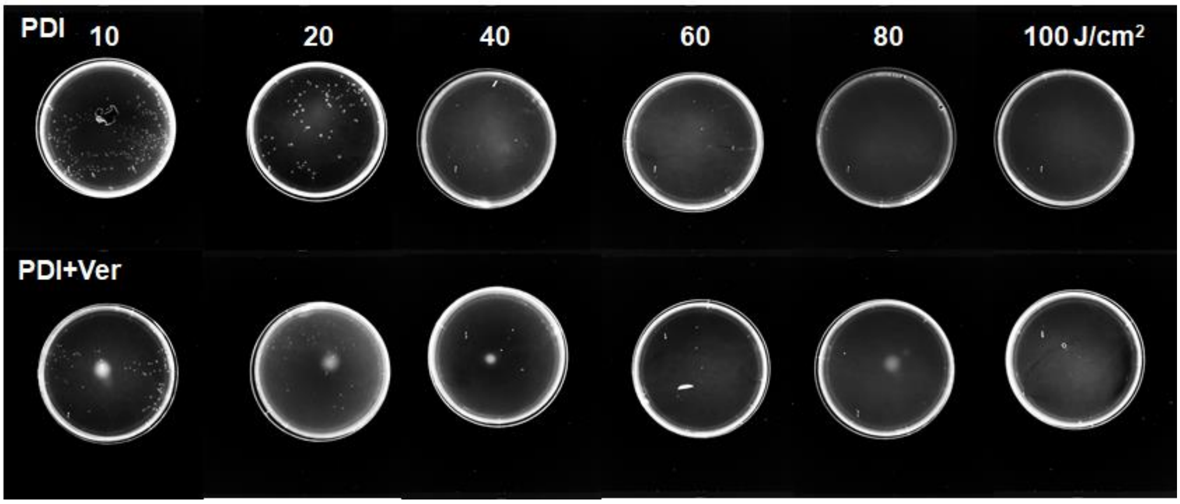

Figure S21. The aliquots samples of *S. aureus* treated with Cl_2TPPS with various light dose 10 - 100 J/cm^2.

Reference

1. Simone, B. C. D.; Mazzone, G.; Russo, N.; Sicilia, E.; Toscano, M. J. M., Metal atom effect on the photophysical properties of Mg (II), Zn (II), Cd (II), and Pd (II) tetraphenylporphyrin complexes proposed as possible drugs in photodynamic therapy. **2017,** 22, (7), 1093.
2. Cook, L. P.; Brewer, G.; Wong-Ng, W. J. C., Structural aspects of porphyrins for functional materials applications. **2017,** 7, (7), 223.
